# Differentiating Alzheimer’s Aβ Isoforms Coaggregated in Cerebrospinal Fluid via Single-Particle Imaging

**DOI:** 10.1021/acschemneuro.5c00692

**Published:** 2026-01-14

**Authors:** Lily Henry, Shayon Bhattacharya, Talia Bergaglio, Dorothea Pinotsi, Peter Niraj Nirmalraj

**Affiliations:** † Transport at Nanoscale Interfaces Laboratory, Swiss Federal Laboratories for Materials Science and Technology, Dübendorf CH-8600, Switzerland; ‡ Department of Biological Sciences, Bernal Institute, 8808 University of Limerick, Limerick V94T9PX, Ireland; § Scientific Centre for Optical and Electron Microscopy, 27219 ETH Zurich, Zürich 8093, Switzerland

**Keywords:** amyloid beta, protein aggregation, atomic force microscopy, fluorescence microscopy, Alzheimer’s disease, molecular dynamics simulations

## Abstract

Amyloid polymorphism can reflect Alzheimer’s disease (AD) stages. This paper demonstrates that amyloid β (Aβ) peptides, primarily Aβ-40 and Aβ-42 (implicated in AD pathology), present in cerebrospinal fluid (CSF), can be differentiated, and their morphology studied in detail using fluorescence-based super-resolution and atomic force microscopy (AFM). An inhibitory effect of Aβ-40 on Aβ-42 protein aggregation, marked by Aβ-40 oligomers colocalizing along the Aβ-42 fibril backbone, was resolved at the single-particle level. Molecular dynamics simulations revealed that coaggregation is modulated by the ionic environment in CSF, where calcium ions form bridges between Glu residues of Aβ-40 and Aβ-42, known to stabilize the fibril structure. This ion-mediated tethering compacts Aβ-40 and kinetically traps the fibril–oligomer interface, thus reducing fibril elongation. The isoform-specific imaging method further allowed us to distinguish Aβ-40 and Aβ-42 aggregates from oligomers to mature fibrils in the CSF of AD patients, and the nanoscopic differences in aggregate sizes were quantified from the AFM topographs. Such a protein characterization approach, which is not limited by analyte size or shape and is capable of fingerprinting Aβ aggregates in CSF, could be used in clinical settings to monitor the progression of Alzheimer’s disease and related pathologies.

## Graphical abstract

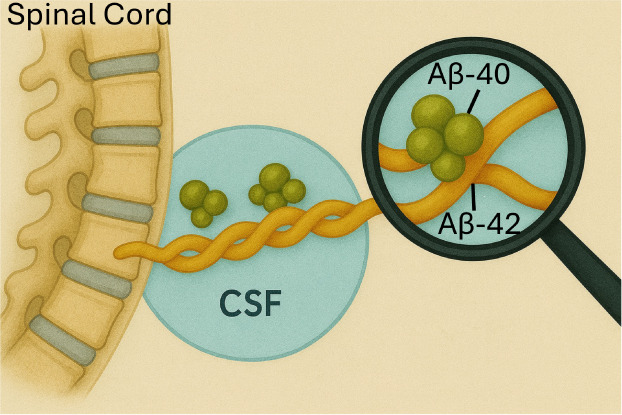

## Introduction

Dementia encompasses a range of symptoms involving memory loss, impaired reasoning, and cognitive decline severe enough to interfere with daily life. It affects approximately 60 million people globally, with projections indicating a 3-fold increase by 2050.^1^ Among these disorders, Alzheimer’s disease (AD) is the most prevalent form, marked by progressive neurodegeneration and deterioration in cognitive and functional abilities. Early presymptomatic detection remains a critical goal in delaying the clinical progression of AD. Currently, cerebrospinal fluid (CSF) biomarkers—primarily amyloid beta (Aβ) and tau proteins—are established indicators of AD pathology and predict disease risk.^2^ While there is growing interest in minimally invasive blood-based biomarkers for identifying cognitive decline,^3^ screening CSF remains the gold standard for early detection in AD, due to its direct interface with the brain and reliable reflection of cerebral Aβ burden. Compared to structural brain imaging, biochemical assays for quantifying proteins in CSF offer a lower-cost and radiation-free option. Notably, amyloid positron emission tomography (PET) imaging, despite being effective in visualizing cerebral Aβ deposition, is often restricted to selected cases due to high operational costs.

The two key Aβ isoforms—Aβ-40 and Aβ-42—are routinely quantified in CSF using immunoassays to monitor AD progression. Of the two, Aβ-40 is typically more abundant, while Aβ-42 is present at approximately 10% of Aβ-40 concentration in brain tissue.^4^ Notably, the soluble oligomeric and protofibrillar forms of Aβ-42 are increasingly recognized as the most neurotoxic species.^5–9^ Aggregation of both peptides begins via primary nucleation pathways^10^ (fibril surface independent), transitioning from monomers to oligomers and protofibrils, eventually forming fibrils and amyloid plaques—hallmarks of AD pathology.^11–13^ These protein aggregates originate in regions such as the entorhinal cortex and hippocampus before progressing to the neocortical association areas of the brain. Despite only a two-residue difference, the presence of two hydrophobic amino acids at the C terminus of Aβ-42 drives its more rapid aggregation compared to Aβ-40.^10,14^ This behavior is attributed to the ability of alanine-42 to form a stabilizing salt bridge with lysine-28, promoting β-sheet formation and fibril stability.^6,15^ In parallel, Aβ-42 aggregation can also proceed via a more accelerated secondary nucleation mechanism^14^—a fibril-surface-dependent pathway influenced by the catalytic properties of seed fibrils and the presence of a small population of superspreading fibrillar species.^11,16^ The primary and secondary aggregation behavior of synthetic Aβ-40 and Aβ-42 peptides has been studied mainly in buffer using thioflavin T (ThT) fluorescence assays,^14,17^ circular dichroism,^18^ atomic force microscopy^10,11^ (AFM), and microfluidics.^19^ Collectively, these studies have elucidated distinct kinetic profiles, aggregation pathways, and size distributions of the resulting amyloid structures. Intriguingly, when Aβ-40 and Aβ-42 peptides are coincubated at equimolar concentrations, Aβ-40 exhibits an inhibitory effect on Aβ-42 fibrillogenesis.^20,20–26^ Although extensive studies in physiological buffers, generally phosphate-buffered saline (PBS), have provided insights into the behavior of coaggregated Aβ-40 and Aβ-42 in human CSF remain scarce, particularly at single-particle resolution. To date, most evidence from CSF has relied on commercially available enzyme-linked immunosorbent assays (ELISA), which lack morphological details of the protein aggregates. High-resolution visualization and quantification of these aggregates in a native CSF milieu under standard laboratory conditions remains a significant gap in our understanding of the early stages of Alzheimer’s disease pathogenesis.

In addition to quantitative analysis and chemical differentiation of Aβ-40 and Aβ-42 levels in CSF, morphological information has also emerged as a valuable diagnostic readout, such as size distribution and shape of protein aggregates (termed “physical biomarkers^27^”). Several nanoscale imaging techniques have demonstrated that differences in protein aggregate morphology reflect stages and severity of neurodegenerative diseases.^27–30^ In previous work from our laboratory, we have demonstrated that the length of individual Aβ fibrils resolved using liquid-based AFM patient-derived CSF correlates with AD progression, from subjective cognitive decline, mild cognitive impairment, to advanced-stage clinical symptoms.^31^ Yet, a key challenge remains: the ability to distinguish coaggregated Aβ-40 and Aβ-42 peptides at single-particle resolution directly in a native CSF biofluid environment under standard laboratory conditions. We posited that a multimodal strategy combining label-free nanoscale imaging (AFM, 3-D digital holotomography) with fluorescence-based super-resolution microscopy could overcome individual limitations and enable both morphological and chemical identification of Aβ-40 and Aβ-42 individual aggregates and coaggregated forms, from oligomers to fibrils, with high biochemical specificity in a mixture below the resolution diffraction limit of typical light microscopy.^32^ AFM offers nanometric resolution of aggregate shape and topology in near-native states but lacks chemical specificity. Conversely, fluorescence methods enable isoform-specific detection via labeled antibodies or dyes, albeit at the cost of potential artifacts, such as dye-induced changes in fibril morphology or oligomer size distribution.^33^ While conducted *in vitro*, these experiments could clarify Aβ-40/Aβ-42 coaggregation mechanisms, as neither isoform aggregates in isolation in brain tissue, and the environment strongly influences their aggregating propensity.^34^ Recent simulation studies on amyloidogenic interfaces, including tau and α-synuclein assemblies, suggest that environmental context, partial disorder, and membrane interactions profoundly modulate aggregation dynamics.^35,36^

Here, we demonstrate that Aβ-40 and Aβ-42 protein aggregates—ranging from oligomers to protofibrils and mature fibrils—can be resolved, quantified, and chemically differentiated under biologically relevant conditions. We incubated equimolar peptide mixtures (1 μM, 37 °C, 48 h, 400 rpm shaking) in human CSF and imaged the resulting aggregates using label-free nanoscale imaging (atomic force microscopy, 3D digital holotomography) and fluorescence-based super-resolution microscopy techniques. To develop a more detailed explanation of the experimental observations, we performed molecular dynamics (MD) simulations by modeling the coaggregation interface between Aβ-40 oligomers and Aβ-42 fibrils under both PBS and CSF-mimicking aqueous ionic environments. Together, the experimental and computational findings highlight a physical basis for Aβ-40-mediated attenuation of Aβ-42 aggregation. Finally, we extend our imaging approach to probe CSF samples from a small cohort (*n* = 5) of AD patients, providing proof-of-principle for detecting and chemically resolving Aβ isoform-specific aggregates directly in clinical samples. These findings provide a promising approach for monitoring disease progression through amyloid isoform-specific aggregate profiling in human biofluids.

## Results and Discussion

### Characterization of Aβ-40 and Aβ-42 Oligomers and Fibrils in PBS Solution

To resolve and distinguish coaggregated states of Aβ-40 and Aβ-42 peptides in complex fluids, such as cerebrospinal fluid (CSF), we first established validated imaging protocols in physiological phosphate-buffered saline (PBS). Aβ-40 and Aβ-42 peptide solutions were independently incubated for 48 h (see Materials and Methods section) in PBS (10 mM, 2.7 mM KCl, 137 mM NaCl, pH 7.4), a condition previously shown by us to yield both oligomers and fibrils during Aβ-40 and Aβ-42 primary aggregation pathways.^10^ Figure 1A shows an AFM recorded after depositing Aβ-40 peptides (incubated for 48 h) on a gold substrate, followed by air-drying, gentle rinsing using pure water, and air-drying before imaging. This sample preparation process ensures that protein aggregates have firmly adhered to the surface and that the excess salt deposits are removed to facilitate artifact-free and high-resolution AFM imaging. The Au(111) substrate grown on mica disks used in this study has been validated from our previous work for high-resolution AFM imaging of protein aggregates, providing atomically flat terraces without denaturing surface-adsorbed species.^31,37^ AFM imaging of Aβ-40 (Figure 1A) revealed both isolated fibrils and a higher prevalence of spherical particles of varying sizes. Height profiles (Figure 1B) extracted along the blue line in Figure 1A, indicate spherical aggregates to be larger in diameter compared to the height of the single fibril. As the height equals the diameter of spherical and cylindrical objects, the measured height profiles (a parameter that is not influenced by tip geometry) can be used to estimate the particle diameter. Although it is possible to estimate particle sizes in a label-free manner, it cannot unambiguously distinguish between proteinaceous oligomeric particles and salt-derived particles, which can stem from the salt residues present in the PBS medium. To confirm the presence of Aβ-40 oligomers, we used indirect immunofluorescence labeling with Alexa Fluor 561 (see Figure S1 in Supporting Information) and imaged the aggregates using super-resolution microscopy—specifically Stochastic Optical Reconstruction Microscopy (STORM) (see Materials and Methods for details on the antibody staining procedure and super-resolution imaging protocols)—a single molecule localization-based method. Super-resolution imaging (Figure 1C) revealed numerous fluorescently labeled Aβ-40 oligomers consistent with the AFM observations, while fibrillar structures were rarely detected, likely due to their low abundance. In contrast, Aβ-42 peptides incubated for 48 h (Figure 1D) formed dense fibrillar networks with prevalent spherical particles. Cross-sectional AFM height profile analysis (Figure 1E, extracted along the blue line indicated in Figure 1D) showed that Aβ-42 fibrils are more elongated and the fibril bundles are larger in diameter compared to Aβ-40 counterparts (Figure 1A), confirming previous reports on higher aggregation propensity of Aβ-42.^10,31^ STORM imaging of Alexa Fluor 647-labeled Aβ-42 aggregates (Figure 1F) confirmed the presence of both fibrillar and oligomeric structures.

**Figure 1:**
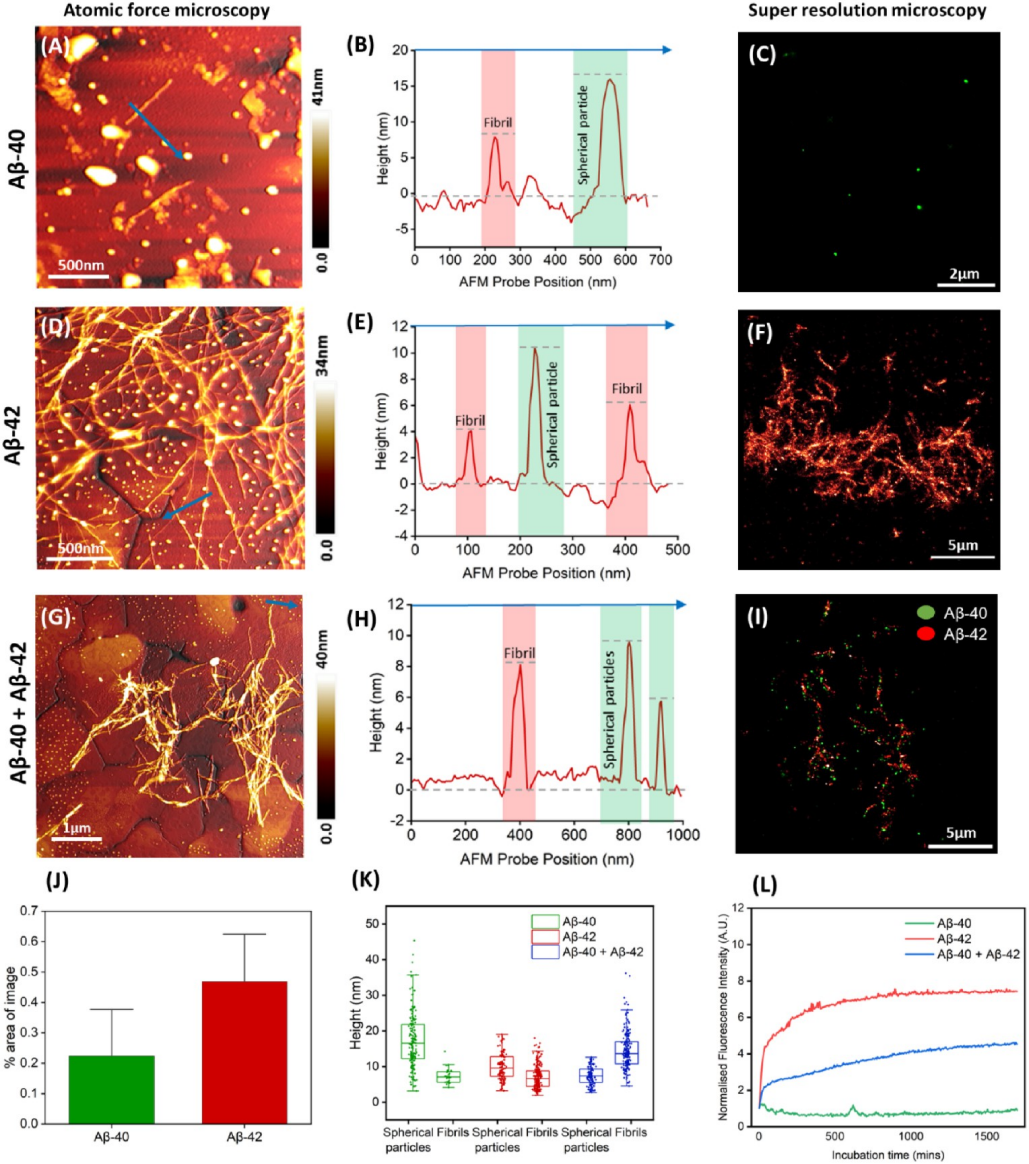
AFM and super-resolution (STORM) imaging of separately and coaggregated Aβ-40 and Aβ-42 peptides incubated in PBS. (A) AFM topographic image showing the presence of spherical particles of varying sizes and short fibrils adsorbed on a gold substrate. (B) Cross-sectional height profile indicated by the blue arrow in panel A. (C) Super-resolution fluorescence microscopy image of Aβ-40 labeled with Alexa Fluor 561 showing the presence of mainly spherical oligomeric particles. Data shown in panels A–C are based on Aβ-40 peptides incubated separately. (D) AFM image of Aβ-42 protein aggregates showing mostly elongated fibrils together with spherical particles. (E) Cross-sectional height profile indicated by the blue arrow in panel D. (F) Super-resolution fluorescence microscopy image of Aβ-42 labeled with Alexa Fluor 647, detecting both fibrillar and oligomeric protein aggregates. Data shown in panels D–F are based on Aβ-42 peptides incubated separately. (G) AFM image of coaggregated Aβ-40 and Aβ-42 showing the presence of both fibrillar and spherical particles. (H) The cross-sectional height profile is indicated by the blue arrow in panel G. (I) Super-resolution fluorescence microscopy image of Aβ-40 labeled with Alexa Fluor 561 in green and Aβ-42 labeled with Alexa Fluor 647 coded in red. (J) Percentage area and standard deviation taken up by signal from each channel in 4 images of coaggregated samples: Aβ-40 (mean = 0.22 ± 0.14%). and Aβ-42 (mean = 0.47 ± 0.14%). (K) Quantification of spherical particle and fibril height for Aβ-40 and Aβ-42 aggregated independently and coaggregated based on AFM images. (L) ThT kinetics assay of the total concentration of 1 μM of peptides in PBS per sample for independent and coaggregated protein samples.

After confirming the aggregated states of Aβ-40 and Aβ-42 incubated separately, a mixed coaggregated solution of Aβ-40 and Aβ-42 (1 μM total peptide concentration) was prepared and incubated for 48 h (see Materials and Methods). AFM images (Figure 1G–H) showed a heterogeneous population of fibrils and spherical aggregates. Figure 1G is a large area scan showing predominantly the formation of fibrils, together with isolated spherical particles adsorbed on the gold surface. The cross-sectional profile (Figure 1G) shows the nanoscopic differences in height between mature fibrils and spherical particles (Figure 1H) resolved in the AFM topography. Dual-color STORM imaging (Figure 1I) revealed distinct colocalization of Alexa Fluor 561-labeled Aβ-40 oligomers (green) with Alexa Fluor 647-labeled Aβ-42 fibrils (red), predominantly along fibrillar backbones. Detailed analysis of the super-resolution image revealed mostly the colocalization of Aβ-40 oligomers with Aβ-42 fibrillar aggregates. Based on the qualitative distribution of Aβ-40 and Aβ-42 protein aggregates visible from the super-resolution image, we quantify the mixed population by calculating the percentage area of the image occupied by the signal obtained from each channel in four independent super-resolution images. Figure 1J is a plot showing the quantitative differences in the composition of Aβ-40 and Aβ-42 protein aggregates resolved using super-resolution microscopy. The combined size distribution plot (Figure 1K) obtained from the AFM measurements of the unlabeled Aβ-40 (green histogram) and Aβ-42 (red histogram) protein aggregates (both oligomers and fibrils) incubated separately indicates that Aβ-42 fibrils are larger in diameter compared to Aβ-40 counterparts. Statistical analysis of size distribution confirmed that while individually incubated Aβ-40 and Aβ-42 differed in both spherical and fibril dimensions, their coaggregated forms (blue histogram), exhibited distinct size shifts, possibly due to intermolecular interactions. A mean spherical particle size of 17.25 ± 7.60 nm and a mean fibril diameter of 7.35 ± 2.15 nm for separately incubated Aβ-40 peptides was observed. The mean spherical particle size of 10.00 ± 3.80 nm and a mean fibril diameter of 7.00 ± 3.30 nm was observed for separately incubated Aβ-42 peptides. The mean spherical particle and fibril diameters for coaggregated Aβ-40 and Aβ-42 peptides were calculated to be 7.40 ± 2.55 nm and 14.40 ± 5.20 nm, respectively. The bars in all histograms indicate the 25th percentile, median, and 75th percentile, plus minimum and maximum values of all spherical particles and fibrils in each specific group.

Together, these data demonstrate that both AFM and super-resolution microscopy can resolve and chemically differentiate Aβ isoforms at the single-particle level. Aβ-42 aggregates more readily than Aβ-40, and selective fluorophores can distinguish their respective aggregates in mixed samples. Yet, to develop a deeper understanding of the protein aggregation process, ensemble-level assays are needed to probe differences in aggregation kinetics. To this end, we performed Thioflavin-T (ThT) fluorescence assays on separately and coaggregated Aβ-40 and Aβ-42 peptides in PBS (see Materials and Methods). ThT is a dye that is commonly used as a molecular probe to monitor the aggregation of amyloidogenic proteins. As shown in Figure 1L, coaggregation with Aβ-40 (blue trace) suppressed Aβ-42 aggregation relative to Aβ-42 alone (red trace), consistent with prior reports.^20^ Complementary FTIR measurements confirmed this attenuation and further revealed beta-sheet signatures in Aβ-40 aggregates formed in CSF but not in PBS (see Figure S2 for details on FTIR measurements).

### Characterization of Aβ-40 and Aβ-42 Protein Aggregates Directly in CSF

To evaluate how aggregation differs in physiologically relevant environments, we extended our PBS-based imaging workflow to commercially available, synthetic human cerebrospinal fluid (CSF) (see Materials and Methods section for details on the composition of synthetic CSF). AFM height maps recorded across multiple regions revealed that Aβ-40 peptides aggregated in CSF form predominantly short, heterogeneous fibrils and spherical particles (Figures 2A–B and S3) in line with previous reports describing Aβ-40s tendency to form structurally heterogeneous fibrils.^38^ Super-resolution microscopy revealed closely spaced Aβ-40 oligomers in CSF (Figure 2C) in contrast to the more dispersed structures observed in PBS (Figure 1C), suggesting medium-specific modulation of oligomer interactions. For Aβ-42 peptides, AFM heights revealed more densely packed fibrils in CSF than in PBS (Figure 2D–E), suggesting CSF could influence how the protein aggregates. Furthermore, super-resolution microscopy revealed highly aggregated structures as shown in Figure 2F, with regions of higher signal intensity (depicted in white) indicating dense fibrils. Quantitative analysis showed Aβ-40 forming larger spherical particles than Aβ-42 in both CSF and PBS (Figures 2G, 1K). These findings point to Aβ-42 aggregation in both media appearing to drive the formation of densely packed fibrillar networks, potentially depleting the pool of smaller oligomers. Further quantitative analysis for the fibril height of each isoform is provided in Figure 2H. We next examined Aβ-40 and Aβ-42 coaggregation in CSF (each at 500 nM, total 1 μM). AFM revealed extensive fibrillar networks, albeit less dense than for Aβ-42 alone (Figure 3A,B), with increased average fibril height consistent with Aβ-40 localizing onto Aβ-42 fibrils (Figure 3C), a phenomenon previously observed in PBS (Figure 1K). This may reflect both the inherently compact fibrils formed by Aβ-42, driven by its two additional C-terminal hydrophobic residues, and the localization of Aβ-40 onto Aβ-42 fibrils, as previously visualized by super-resolution microscopy in PBS. Such mixed assemblies are supported by prior reports of Aβ-40/Aβ-42 coaggregation,^39,40^ and NMR has shown Aβ-40 monomers have a higher affinity to bind to aggregates of Aβ-42 compared to Aβ-42 monomers, with the two isoforms competing to bind to preexisting aggregates.^41^ STORM imaging confirmed Aβ-40 colocalization on Aβ-42-rich fibrils (Figures 3D–F) with Aβ-42 comprising the majority of the aggregate mass (2.55% vs 0.17%, Figure 3G), a finding consistently confirmed across four independent images. These data suggest Aβ-42 dominates the aggregation landscape even under equimolar conditions, with a stronger bias in CSF than in PBS (Figure 1J), while still allowing for the incorporation of Aβ-40 into mixed assemblies. Super-resolution and other fluorescence-based imaging of Aβ isoforms have previously been employed to investigate Aβ-40 and Aβ-42 individually in vitro and in vivo.^16,42–45^ The present study extends this approach by applying STORM to examine the two isoforms concurrently within body fluid-derived samples, enabling direct visualization of their spatial organization and coaggregation. This approach offers additional relevance in the context of clinically accessible biofluids and provides mechanistic insight into protein interactions under near-physiological conditions. While morphological observations provide insight into how protein aggregation physically differs in CSF compared to PBS, evidence of the influence CSF on aggregation of the isoforms when aggregated independently and coaggregated is needed to fully understand the underlying mechanisms observed through imaging. Thus, to probe the kinetics underlying this behavior, we conducted a ThT-based aggregation assay in CSF (Figure 3I), as done previously in PBS. Changes in ThT fluorescence intensity were tracked for Aβ-40 (green), Aβ-42 (red), and coaggregated Aβ-40 and Aβ-42 (blue), at a protein concentration of 1 μM. Aβ-42 aggregation was suppressed by coaggregation with Aβ-40, consistent with PBS, but Aβ-40 also showed delayed yet increased ThT signal in CSF—a feature absent in PBS—indicating enhanced β-sheet formation. This was supported by FTIR data, which revealed a β-sheet signature for Aβ-40 in CSF not seen in PBS (Figure S2). These results suggest CSF stabilizes fibril-prone structures and facilitates the conversion of oligomers into mature aggregates, particularly for Aβ-42.

**Figure 2:**
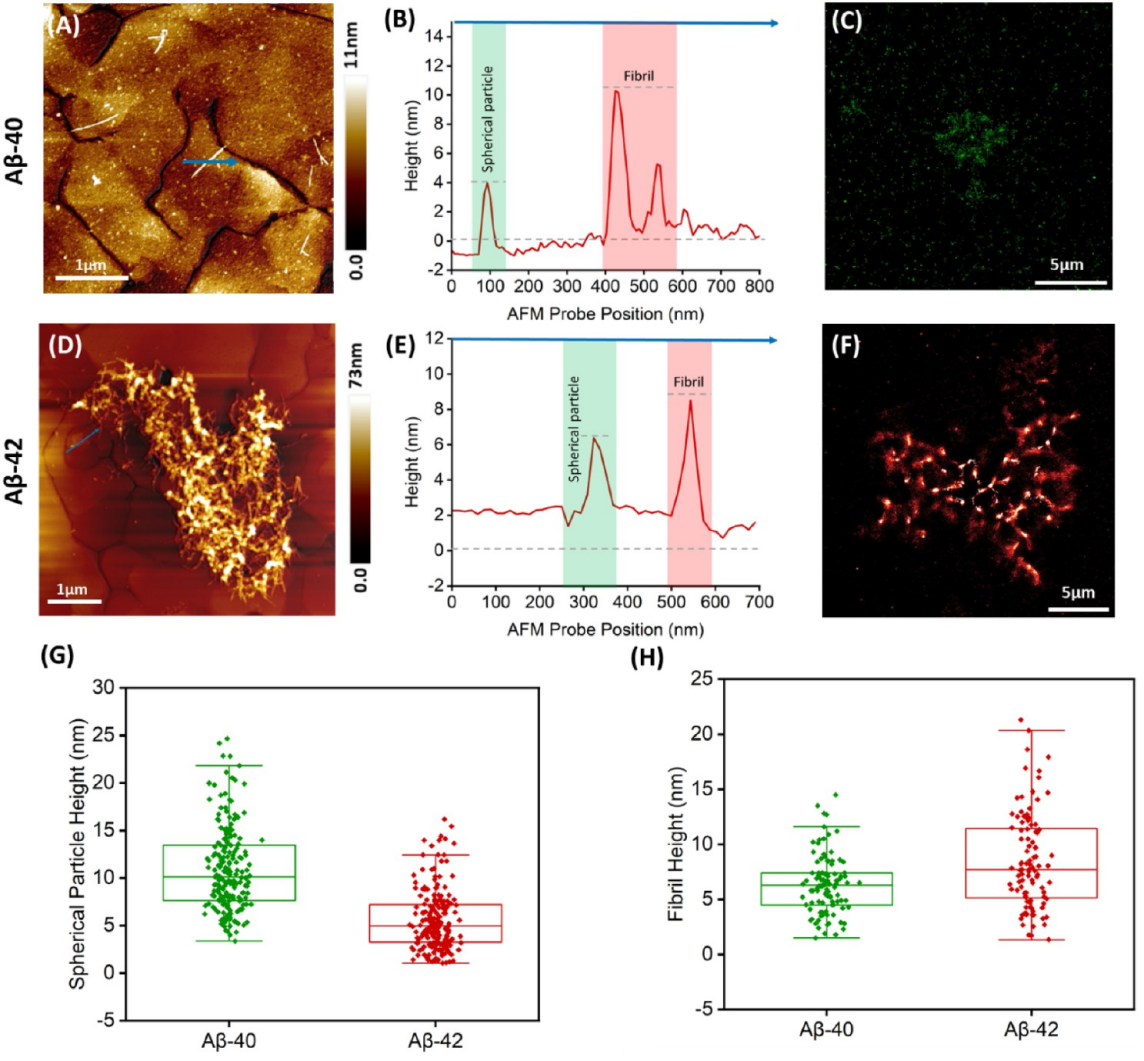
AFM and super-resolution analysis of separately incubated Aβ-40 and Aβ-42 protein aggregates in synthetic human CSF. (A) AFM topographic image of spherical particles of Aβ-40 on a gold substrate. (B) Cross-sectional height profile indicated by the blue arrow in (A). (C) Super-resolution fluorescence microscopy image of Aβ-40 labeled with Alexa Fluor 561 showing the presence of protein oligomers. (D) AFM topographic image of dense Aβ-42 fibrillar aggregates on gold substrate. (E) Cross-sectional height profile indicated by the blue arrow in (D). (F) Super-resolution fluorescence microscopy image of Aβ-42 labeled with Alexa Fluor 647 showing fibrillar protein aggregates. (G) Quantification of spherical particle height for Aβ-40 and Aβ-42 based on AFM topographical images: Aβ-40 (*n* = 198, mean = 10.94 ± 4.38 nm), Aβ-42 (*n* = 210, mean = 5.62 ± 3.08 nm). (H) Quantification of fibril height for Aβ-40 and Aβ-42 based on AFM topographical images: Aβ-40 (*n* = 103, mean = 6.3 ± 2.65 nm), Aβ-42 (*n* = 100, mean = 8.46 ± 4.34 nm). Bars indicate the 25th percentile, median, and 75th percentile plus minimum and maximum values of all spherical particles and fibrils in each group.

**Figure 3:**
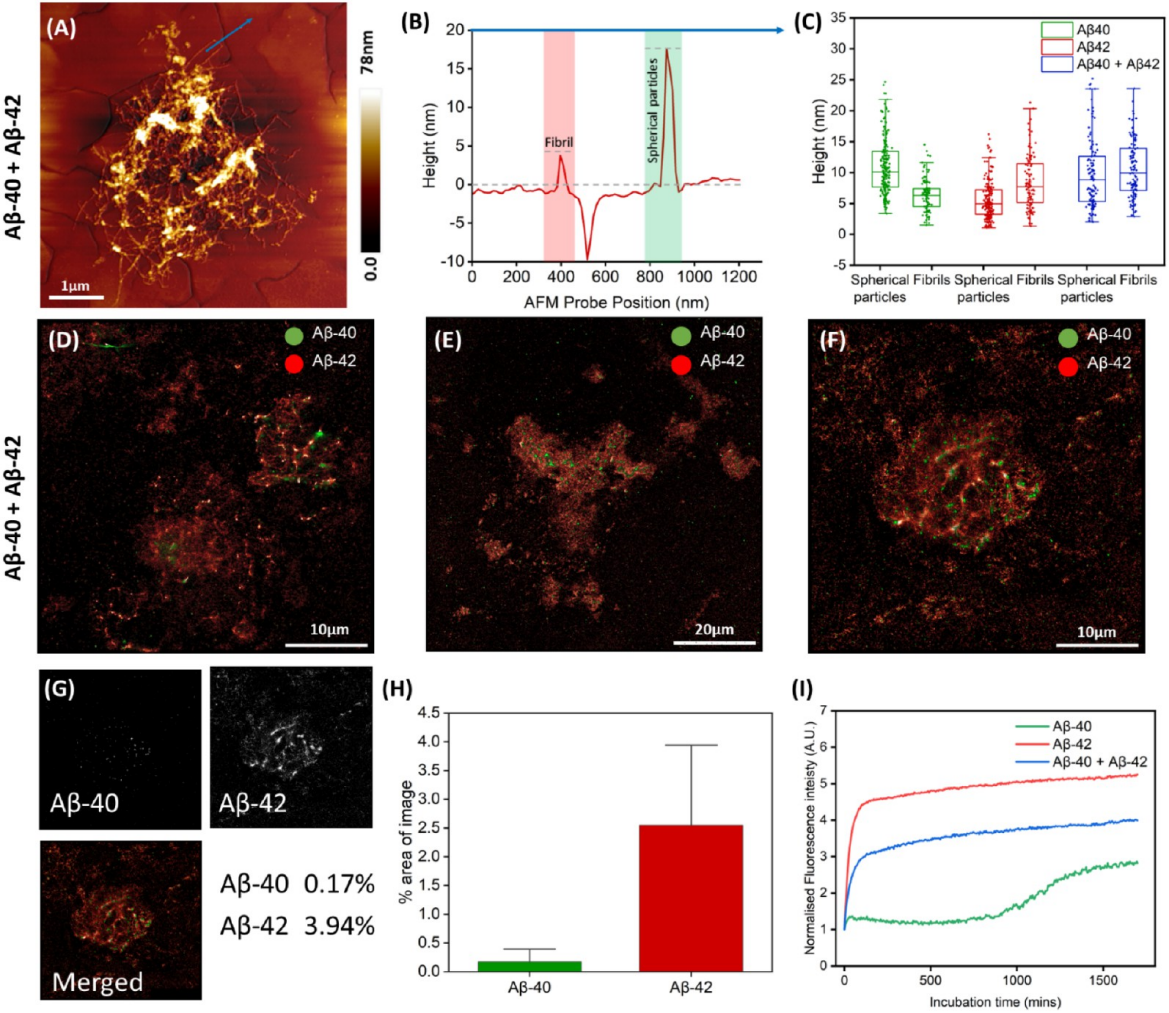
AFM and super-resolution analysis of Aβ-40 and Aβ-42 protein aggregates in CSF when aggregated together. (A) AFM topographic image of fibrillar aggregate adsorbed on gold substrate. (B) Cross-sectional height profile is indicated by blue arrow in (A). (C) Quantification of spherical particle and fibril height for Aβ-40 and Aβ-42 aggregated independently and coaggregated based on AFM topographical images: Aβ-40 spherical particles (*n* = 198, mean = 10.94 ± 4.38 nm) and fibrils Aβ-40 (*n* = 103, mean = 6.3 ± 2.65 nm), Aβ-42 spherical particles (*n* = 210, mean = 5.62 ± 3.08 nm) and fibrils (*n* = 100, mean = 8.46 ± 4.34 nm), Aβ-40 + Aβ-42 spherical particles (*n* = 110, mean = 9.77 ± 5.56 nm) and fibrils (*n* = 112, mean = 10.65 ± 4.79 nm). Bars indicate the 25th percentile, median, and 75th percentile, plus minimum and maximum values of all spherical particles in each group. (D, E, F) Super-resolution fluorescence microscopy images of Aβ-40 labeled with Alexa Fluor 561 in green and Aβ-42 labeled with Alexa Fluor 647 in red, showing large aggregates of Aβ-42 containing Aβ-40 oligomers. (G) Super-resolution images processed with ImageJ showing binary images for each protein channel after threshold filtering. Area fraction measurements show the percentage of the image occupied by signal for each channel. (H) Percentage area and standard deviation taken up from signal in each channel in 4 images of coaggregated samples: Aβ-40 (mean = 0.17 ± 0.16%) and Aβ-42 (mean = 2.55 ± 1.21%). (I) ThT kinetics assay of total concentration of 1 μM in CSF per sample (0.5 μM per protein for coaggregated samples) for independent and coaggregated protein samples.

Together, these findings support our earlier interpretation of the influences of CSF on the aggregation kinetics and structural rearrangements of Aβ. This suggests that more stable structures are formed in CSF, consistent with higher beta-sheet content being associated with stable oligomers and fibrils.^46^ Interestingly, previous work has shown that during the primary nucleation pathway of Aβ-42, the majority of oligomers dissociate back into monomers, rather than aggregating further into fibrils.^47^ The increase in fibrillar aggregates following aggregation in CSF compared to PBS, therefore, suggests that the medium itself promotes the stabilization of aggregates and their conversion to more mature aggregate structures. The data underscore the influence of ionic composition on Aβ aggregation and demonstrate the value of characterizing both independent and coaggregation of amyloid peptides under near-physiological conditions.

### Molecular Simulations Reveal Ion-Specific Interfacial Tethering in Aβ-40–Aβ-42 Coaggregates

To investigate the molecular basis of Aβ-40–Aβ-42 coaggregation observed in our AFM experiments, we performed molecular dynamics (MD) simulations of docked Aβ-40 nonamer^48^ (oligomer composed of 3-fold trimer) on a 24-mer Aβ-42 protofibril^49^ (see Molecular Modeling and Dynamics Simulations in SI) under PBS and CSF environments. Among the top-scoring docked poses generated using ZDOCK,^50^ Model 2 (Figure S4A, red box) was selected for subsequent simulations, consistent with AFM-observed surface adhesion. In contrast, Model 1, similar in docking energy, positioned the Aβ-40 oligomer along the Aβ-42 fibril elongation axis, a configuration not supported by experimental evidence, likely reflecting the discrete, finite model size. This model, along with the fibril–oligomer complex, was adsorbed onto an Au(111) slab (Figure  S4C, top view) to mimic experimental imaging and solvated in PBS or CSF ionic environments (Figure  S4B). Each system was simulated for 300 ns. Convergence of the simulations was confirmed via the fraction of native contacts *Q*(*X*) (Figure  S4D), with both systems showing plateaued behavior. Compaction of the coaggregates was then quantified from trajectory-derived height distributions relative to the gold surface (Figure 4A), revealing a statistically significant shift toward lower heights in CSF, indicating closer oligomer-fibril-substrate packing and the trend consistent with AFM height measurements. This observation was reinforced by the reduced sphericity (Δ, see SI for Molecular Modeling and Dynamics Simulations) of the Aβ-40 oligomer in CSF (Figure 4B), indicating a more symmetric, compact shape. However, the time-resolved radius of gyration (*R*_g_) analyses (Figure S4E) showed equal *R*_g_ drop in both environments for the final 100 ns, hinting at compaction of oligomers. This finding suggests that oligomers are equally compact in both PBS and CSF overall, but slightly more isotropic (less elongated) in CSF. To dissect the interaction determinants, we decomposed the oligomer–fibril interaction energy by domain (Figure 4C). In PBS, slightly stronger interactions were observed between the Aβ-40 N-terminus and both the N-terminal and C-terminal regions of Aβ-42 fibril than in CSF, with minor contributions from Aβ-40 N-terminus and Aβ-42 central hydrophobic cluster (CHC) interactions in both environments. In addition, in CSF, prominent Aβ-40–Aβ-42 inter-C-terminus interaction was observed. Time-resolved energy decompositions (Figure  S4H–I) support this, showing greater fluctuations and reduced oligomer–fibril anchoring in CSF, especially during the final 150 ns in PBS, where the oligomer–fibril electrostatic interactions significantly improve in PBS (Figure S4F–G).

**Figure 4:**
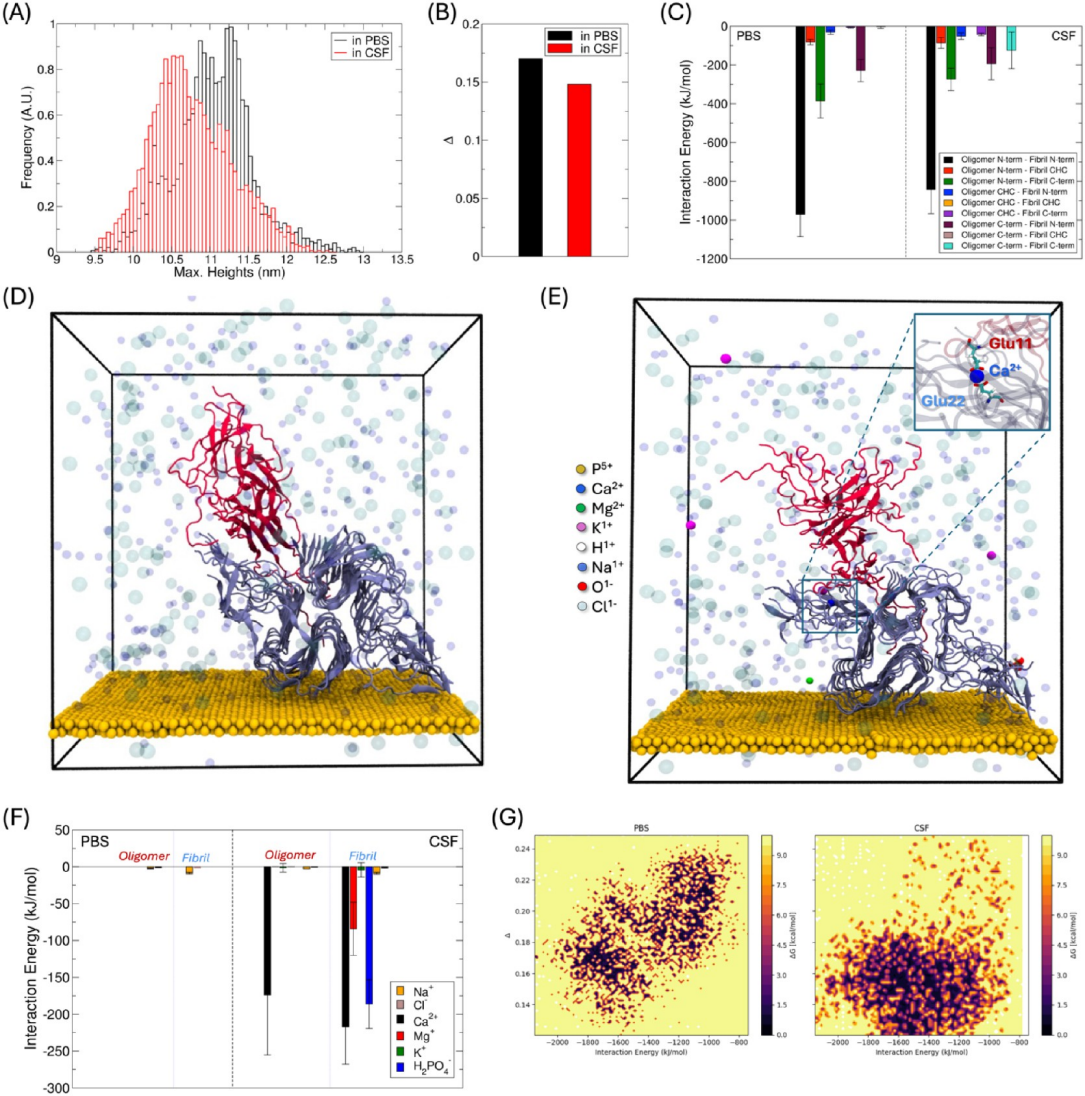
Ion-specific modulation of Aβ_40_–Aβ_42_ coaggregate morphology and energetics in PBS versus CSF. (A) Distribution of maximum vertical heights of the coaggregated complex relative to the gold surface in PBS (black) and CSF (red), extracted from the final 100 ns of simulations. A marked leftward shift in CSF indicates reduced oligomer protrusion and enhanced surface adhesion. (B) Average asphericity (Δ) of Aβ_40_ oligomer, showing increased sphericity in CSF, consistent with morphological compaction. (C) Domain-resolved interaction energy decomposition between the Aβ_40_ oligomer and the Aβ_42_ fibril for both PBS and CSF conditions, highlighting altered binding patterns across fibril regions (N-term, CHC, C-term). (D) Representative snapshot of the Aβ_40_–Aβ_42_ complex in PBS at the gold–solution interface. Aβ_40_ is shown in red, Aβ_42_ in blue, ions as spheres, and the Au(111) surface in yellow. (E) Representative snapshot in CSF, showing tighter packing of Aβ_40_. Inset reveals persistent bridging of a Ca^2+^ ion between Glu11 (Aβ-40) and Glu22 (Aβ-42), stabilizing the interface. (F) Oligomer–ion and fibril–ion interaction energies (normalized per ion) in PBS and CSF, showing significantly stronger binding of Ca^2+^ to both components in CSF. (G) Two-dimensional free energy surface (FES) plots for Aβ-40 asphericity versus total interaction energy in PBS (left) and CSF (right), illustrating distinct morphodynamic regimes: broad, deep-binding states in PBS vs compact, kinetically trapped states in CSF.

To rationalize the observed overall oligomer–fibril interaction energies being more favorable in PBS, we computed ion–peptide interaction energies. In CSF, persistent binding of Ca^2+^ to acidic side chains of Aβ-40 oligomer resulted in significant ion–oligomer interactions (Figure 4F, right; Figure S4K), effectively sequestering the trimer into a compact, electrostatically stabilized conformation. These calcium–oligomer interactions, absent in PBS (Figure S4J), are exemplified by a Ca^2+^ ion acting as an interfacial bridge between two Glu residues of Aβ-40 (Glu11) and Aβ-42 (Glu22), stabilizing the adsorbed compact state (see Figure 4E, inset). Similar ion–fibril interactions (including Mg^2+^) were also stronger in CSF (Figures 4F; Figure  S4M vs Figure  S4L), supporting the idea that calcium competitively screens oligomer–fibril binding by creating alternative stabilizing contacts in CSF. This ion-mediated effect may alter local fibril packing or inhibit fibril-stabilizing canonical salt-bridge formation with Glu22, especially under CSF ionic conditions. This ion-mediated cross-binding via Glu11–Ca^2+^–Glu22 could locally disrupt fibril-stabilizing intrafibrillar Glu22–Lys16 salt-bridge^51,52^ or perturb β-arch packing, suggesting that Aβ-40 binding under CSF conditions may promote a kinetically arrested state of Aβ-40 fibrils, potentially off-pathway to elongation. Control simulations in CSF without the Au(111) surface (Figure S5) also show Ca^2+^ engaging acidic residues on Aβ_40_ and Aβ-42 and forming transient Glu/Asp–Ca^2+^–Glu/Asp bridges. These substrate-free simulations confirm that the ion-mediated tethering originates from the CSF-like ionic environment, while the Au(111) interface primarily stabilizes and localizes the coaggregate under experimental conditions. Finally, two-dimensional free energy surfaces (FES) plotted as sphericity versus oligomer–fibril interaction energy (Figure 4G) revealed distinct conformational basins. In PBS, the oligomer sampled extended shapes with deeper binding minima, whereas CSF trajectories populated compact, moderately bound states, consistent with ion-mediated kinetic trapping. Our predictive models collectively demonstrate that the CSF ionic milieu promotes compact morphologies through calcium-mediated stabilization, at the expense of strong oligomer–fibril interaction, while PBS supports more extensive direct binding.

### Label-Free Holographic Imaging of Aβ-40 and Aβ-42 Protein Aggregates in Synthetic CSF

To complement our AFM and STORM analysis, we employed digital holo-tomographic microscopy (DHTM) to interrogate Aβ-40 and Aβ-42 aggregates in synthetic CSF under native, label-free conditions. DHTM offers three-dimensional (3D) refractive index (RI) mapping of samples at nanoscale resolution using low-power laser interference, enabling morphometric quantification of protein aggregates in aqueous environments without fluorescent labeling or drying artifacts (see Materials and Methods for operational details). This approach also enables liquid-state morphometric analysis without the influence of fluorescent labels and washing steps, allowing a comparison of aggregate morphology between dried samples for AFM and those analyzed using super-resolution microscopy. By capturing three-dimensional refractive index distributions, DHTM provides volumetric insights into aggregate morphology and spatial organization, which is particularly valuable when comparing aggregates formed under different conditions or in the presence of potential modulators, such as coaggregation scenarios. This approach has previously been validated for real-time subvisible aggregate tracking using holographic video microscopy of protein solutions^53^ and has been used previously by us for visualizing clot structures and red blood cell morphology under pharmacological perturbation.^54^ Additionally, we have shown that DHTM can reveal ibuprofen-induced morphological changes in red blood cells, highlighting its sensitivity to subtle structural alterations.^55^ Here, we applied DHTM to capture the volumetric morphology and RI distribution of Aβ-40, Aβ-42, and their coaggregated forms in synthetic CSF in a label-free manner. Representative 3D RI reconstructions of Aβ-40 (Figure 5A) revealed numerous small and midsized aggregates, further segmented and highlighted in red in Figure 5B. In contrast, Aβ-42 (Figure 5C–D) yielded larger, denser aggregates under identical conditions.

**Figure 5:**
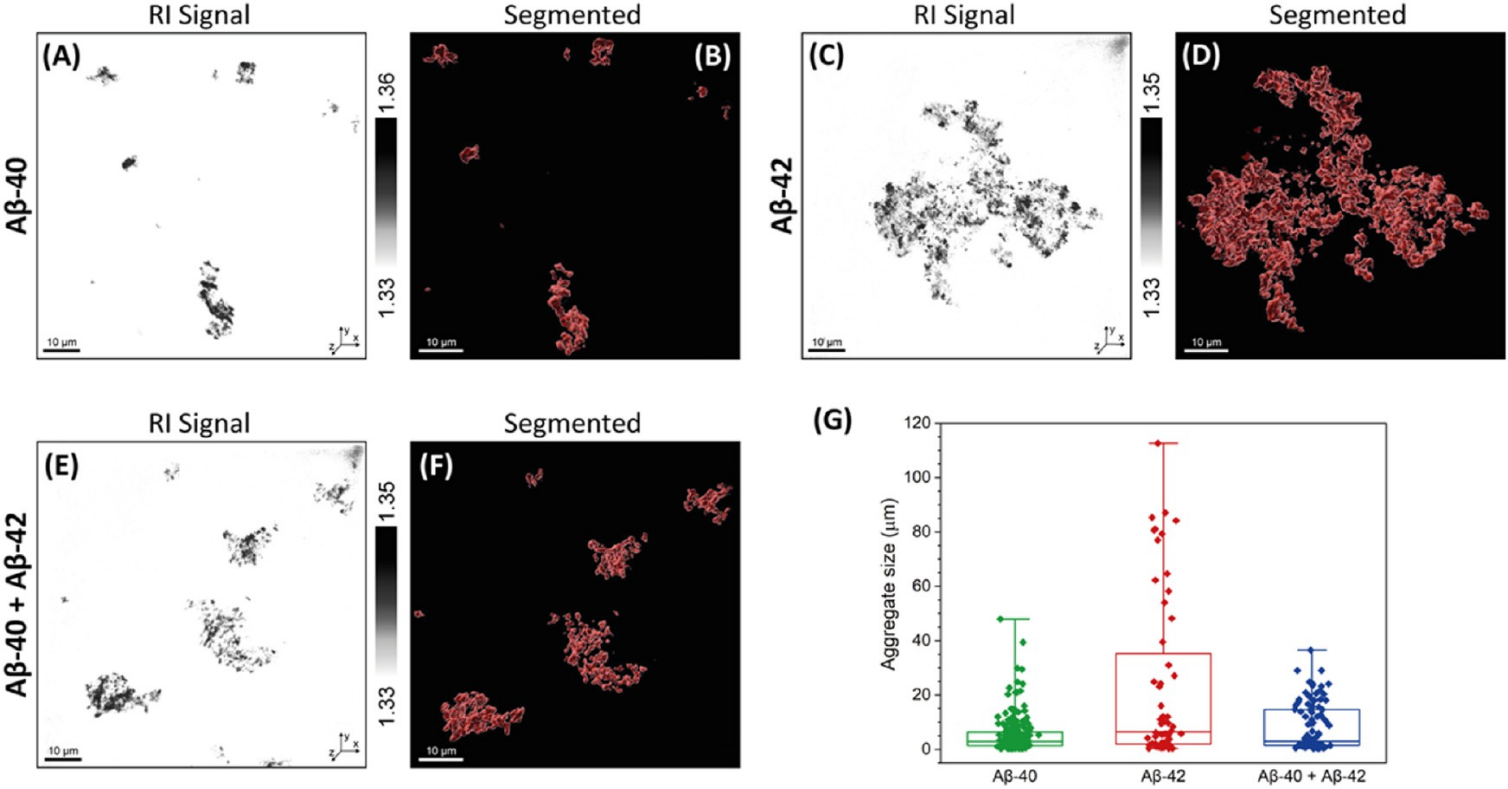
Structural analysis of Aβ-40, Aβ-42, and Aβ-40 + Aβ-42 protein aggregates in CSF. (A) Refractive index (RI) tomogram and (B) corresponding segmented RI tomogram of Aβ-40 protein aggregates in CSF. (C) Refractive index (RI) tomogram and (D) corresponding segmented RI tomogram of Aβ-42 protein aggregates in CSF. (E) Refractive index (RI) tomogram and (F) corresponding segmented RI tomogram of Aβ-40 + Aβ-42 protein aggregates in CSF. (G) Quantification of protein aggregate size variations in CSF: Aβ-40 (*n* = 219, mean = 5.12 ± 6.62 μm), Aβ-42 (*n* = 56, mean = 23.49 ± 30.77 μm) and Aβ-40 + Aβ-42 (*n* = 106, mean = 7.91 ± 8.55 μm). Bars indicate 25th percentile, median, and 75th percentile plus minimum and maximum values of all counted protein aggregates in each group.

Co-incubated samples of Aβ-40 and Aβ-42 peptides (Figure 5E–F) displayed morphologically distinct populations, appearing less extensive than Aβ-42 alone but more heterogeneous than Aβ-40 alone. Following the qualitative DHTM imaging shown in Figure 4A–F, Figure 4E is a combined quantitative plot showing the differences in sizes of Aβ-40, Aβ-42 when aggregated separately and together in CSF medium. Quantitative DHTM analysis (Figure 5G) confirmed these observations: Aβ-42 aggregates exhibited the largest size distribution (mean 23.49 ± 30.77 μm), consistent with its stronger aggregation propensity seen in AFM and super-resolution microscopy. In contrast, Aβ-40 aggregates were significantly smaller (5.12 ± 6.62 μm) compared to Aβ-42 aggregates (23.49 ± 30.77 μm), confirming the previous AFM and super-resolution measurements that Aβ-42 tends to aggregate into higher-order structures compared to Aβ-40 when incubated under identical conditions and periods. Notably, the coaggregated of Aβ-samples yielded intermediate-sized structures (7.91 ± 8.55 μm) (Figure 5C–G), supporting an inhibitory effect of Aβ-40 on Aβ-42 fibrillogenesis in CSF. This observation aligns with our earlier imaging and kinetic analyses and reinforces the idea that Aβ-40 limits the formation of higher-order Aβ-42 structures during coaggregation. These findings highlight the utility of DHTM in resolving amyloid aggregate heterogeneity under native conditions and underscore the inhibitory role of Aβ-40 in modulating Aβ-42 aggregation pathways in biologically relevant media.

### Extension of the Imaging Workflow to Distinguish Amyloids in the CSF of Alzheimer’s Patients

Finally, to characterize and chemically differentiate amyloid aggregates in clinical samples, we analyzed CSF from a small cohort of 4 patients with AD and 1 patient with mild cognitive impairment obtained from commercial sources (BioIVT Inc.). Anonymized clinical metadata for each patient—including age, sex, ethnicity, Mini Mental State Examination (MMSE) scores and baseline concentrations of Aβ-40, Aβ-42 and total tau proteins in the CSF of the patients obtained from the clinical team at BioIVT are summarized in Figure 6A. As shown schematically in Figure 6B–C, the hallmark pathological features of AD—amyloid plaques and neurofibrillary tangles—are composed of aggregated Aβ and tau proteins in the brain tissue. Conversely, the differences in the respective size and shapes of these pathological proteins, such as monomers, dimers, trimers, oligomers, protofibrils, fibrils, and plaques (Figure 6C), which could also contain information on disease progression, remain mostly understudied, despite their potential to serve as physical biomarkers of disease progression. Using AFM and widefield fluorescence imaging, we examined the morphology and isoform composition of Aβ-40 and Aβ-42 aggregates in patient-derived CSF. Three representative patients (ID: 8192, 8207, 8822) are presented in the main figures, while data for the remaining two patients (ID: 8153, 8262) are shown in Supplementary Figure S7. AFM topography of patient 8192 (Figure 7A) revealed dense fibrillar networks interspersed with spherical particles in the CSF. Height profile analysis (Figure 7B, extracted from the cross-sectional profile along the blue line indicated in Figure 7A) shows the height differences of the close-packed fibrils with respect to the underlying gold surface. We classified the elongated structures detected in the CSF of patient 8192 as fibrils and not specifically as protofibrils due to the absence of the nodular morphology previously associated with protofibrils.^31,56^ In contrast, patient 8207 CSF showed prominent annular-shaped structures (∼2.5 nm height) embedded among spherical particles (Figure 7C–D).

**Figure 6:**
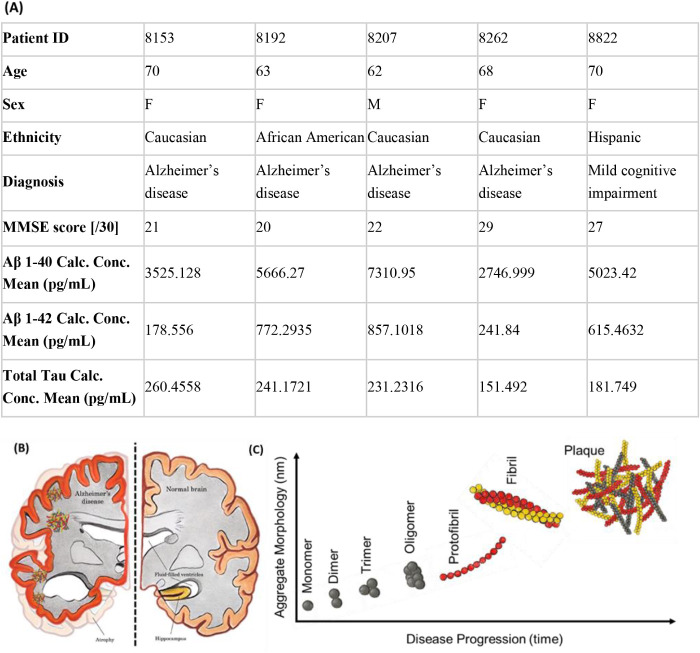
BioIVT AD patient cohort CSF data. (A) Details on patient age, sex, ethnicity, clinical diagnosis, and mini-mental state exam (MMSE) scores of the patients recruited at BioIVT. The amyloid and tau content quantified in CSF using biochemical assays for the 5 patients is also provided in the table. (B) Schematic of protein aggregates in AD brain tissue compared to healthy brain tissue. (C) Schematic showing the morphological changes for progressive amyloid aggregation in Alzheimer’s disease.

**Figure 7:**
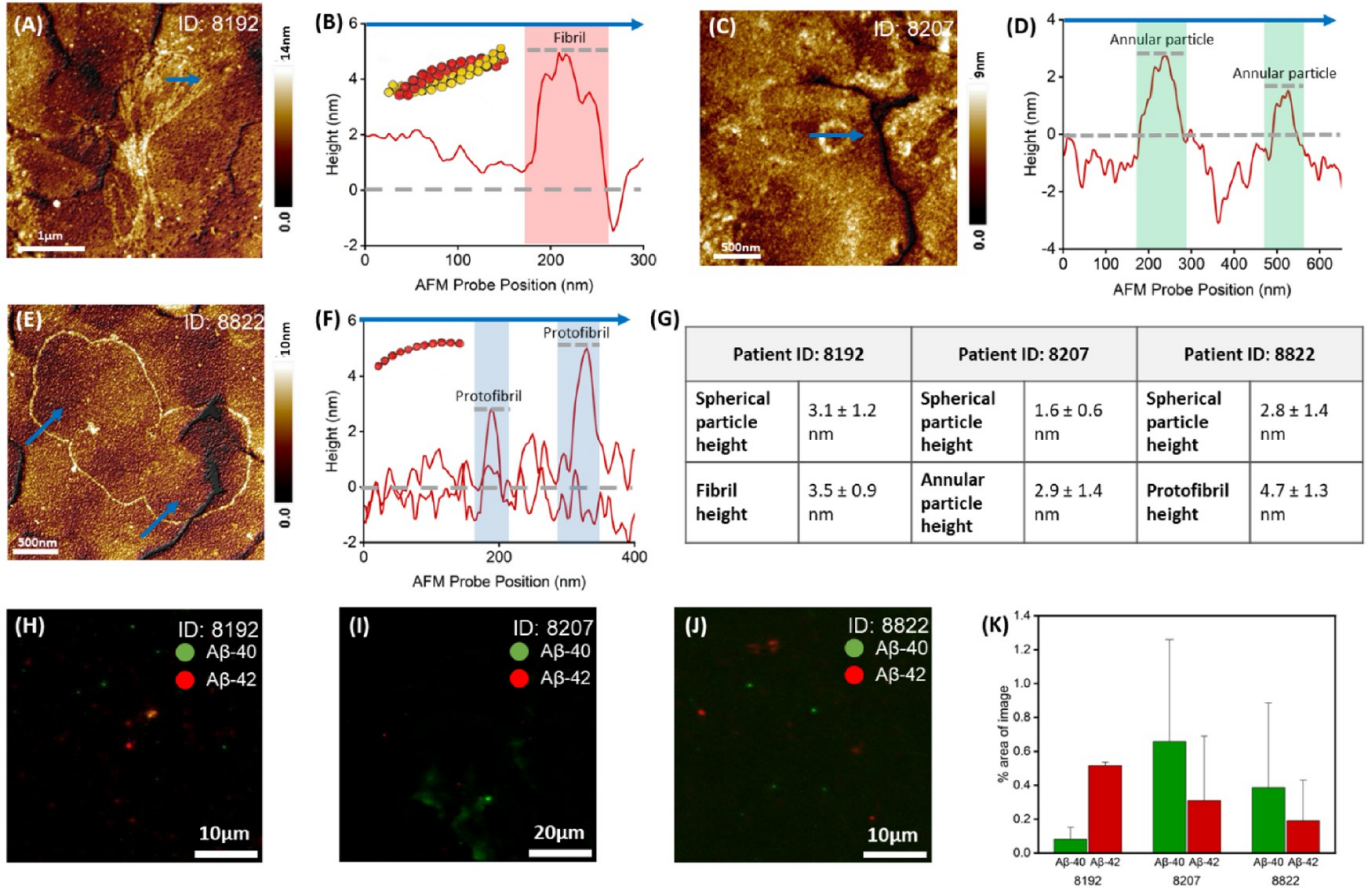
AFM and fluorescence microscopy analysis of Alzheimer’s disease patient-derived CSF samples. (A) AFM topographic image of a fibrillar aggregate surrounded by spherical particles adsorbed on a gold substrate. (B) The cross-sectional height profile is indicated by the blue arrow in (A). (C) AFM topographic image of a nodular annular particle surrounded by spherical particles on a gold substrate. (D) The cross-sectional height profile is indicated by the blue arrow in (C). (E) AFM topographic image of ultralong protofibril surrounded by spherical particles on a gold substrate. (F) Cross-sectional height profiles indicated by blue arrows in (E). (G) Quantification of aggregates identified in AFM topographical images: Patient ID: 8192 spherical particles (*n* = 106, mean = 3.1 ± 1.2 nm) and fibrils (*n* = 97, mean = 3.5 ± 0.9 nm), Patient ID: 8207 spherical particles (*n* = 87, mean = 1.6 ± 0.6 nm) and annular particle (*n* = 14, mean = 2.9 ± 1.4 nm), Patient ID: 8822 spherical particles (*n* = 106, mean = 2.8 ± 1.4 nm) and protofibrils (*n* = 103, mean = 4.7 ± 1.3 nm). (H, I, J) Wide-field fluorescence images of Aβ-40 labeled with Alexa Fluor 561 in green and Aβ-42 labeled with Alexa Fluor 647 in red for patients 8192, 8207, and 8822. (K) Percentage area and standard deviation of image taken up from signal in each channel in 3 images of each patient sample: Patient ID: 8192 Aβ-40 (mean = 0.08 ± 0.07%) and Aβ-42 (mean = 0.5 ± 0.02%), Patient ID: 8207 Aβ-40 (mean = 0.7 ± 0.6%) and Aβ-42 (mean = 0.3 ± 0.4%), and Patient ID: 8822 Aβ-40 (mean = 0.4 ± 0.5%) and Aβ-42 (mean = 2 ± 0.2%).

These annular-shaped forms have previously been detected both in blood-derived AFM data^27^ and in cryo-EM reconstructions of Aβ aggregates along the primary aggregation pathway (∼3 nm height, 8–9 nm diameter).^57^ CSF from patient 8822, diagnosed with mild cognitive impairment in the AD cohort, revealed elongated protofibrils with height variation between 2.5 and 5.5 nm (Figure 7E–F; high-resolution nodular structure in Figure S6). These morphological profiles are consistent with earlier liquid-based AFM studies showing that fibril length correlates with AD disease stage.^31^ More recently, fibrils with unique dimeric nodular units were resolved in the CSF of AD patients, also using AFM.^58^ These studies underscore the benefits of screening CSF from patients with memory and cognitive deficits using high-resolution label-free imaging techniques operating under standard laboratory conditions, as it provide new insights into the role of protein aggregation and disease progression. Note: DHTM measurements were not conducted on the CSF samples from AD patients, as the structures made visible using AFM were below the detection limit of the DHTM tool (spatial resolution ∼ 500 nm). The full spectrum of the aggregates detected using AFM in the CSF of the three patients was quantified and summarized in Figure 7G. Figure S7 in the Supporting Information section shows the AFM images recorded in CSF and the corresponding quantitative analysis of all particles detected in the CSF of the remaining two patients analyzed (8262 and 8153). STORM imaging of these AD patient CSF samples was not feasible due to rapid photobleaching, likely driven by small aggregate size and low epitope density. This is a limitation of STORM in fragile or dilute biological samples due to the high laser intensities over prolonged acquisition times, and fixed fluorophore availability, all of which constrain the accumulation of sufficient localizations for accurate image reconstruction.^59,60^ Nonetheless, using our widefield fluorescent microscopy with optimized immunolabeling protocol, we successfully visualized and differentiated Aβ-40 and Aβ-42 aggregates in all five patient CSF samples (Figures 7H–J and S7). Fluorescence signal was dominated by globular aggregates with two patients showing higher image occupancy for Aβ-40 than Aβ-42 (Figure 7K), mirroring the expected clinical biomarker trend of decreased Aβ-42 levels relative to Aβ-40.^61^ This was also observed in the additional two patient samples analyzed (8153 and 8262, Figure S7). The findings of the current study establish proof-of-concept for high-resolution, label-free, and fluorescence-based differentiation of amyloid isoforms in clinical CSF samples and support the potential of aggregate morphology as a complementary biomarker in AD diagnostics. We anticipate increasing the size of the patient cohort in future studies aimed at differentiating between Aβ-40 and Aβ-42 protein aggregates in CSF medium.

## Conclusion

Using AFM and fluorescence microscopy with isoform-specific antibodies, we demonstrate an approach to clearly distinguish between Aβ-40 and Aβ-42 coaggregated in the cerebrospinal fluid. We provide evidence supporting a coaggregation mechanism in which Aβ-40 oligomers associate with Aβ-42 fibrils, observed from isoform colocalization in dual channel super-resolution microscopy experiments. Importantly, our experiments reveal that the surrounding ionic environment influences aggregation behavior, with enhanced fibril formation in CSF compared to PBS. We anticipate that the protein imaging methodology described in this paper can not only be used to monitor AD progression in CSF but also to study the effect of drugs prescribed such as Levodopa and Lecanemab, to treat neurological diseases directly in CSF medium,^62^ as it mirrors well the pathological changes in the brain.

Complementing these observations, molecular dynamics simulations provide atomic-level insight into how the CSF ionic milieu sculpts coaggregation. In CSF, Ca^2+^ ions mediate persistent electrostatic bridges between Glu11 of Aβ-40 and Glu22 of Aβ-42—residues critical to fibril stability—leading to a more compact, kinetically trapped coaggregate state. The results from the simulations suggest an ion-mediated mechanism of fibril interface stabilization and elongation. Together, our integrative experimental and computational framework uncovers how cerebrospinal fluid composition regulates Aβ isoform interplay and aggregate morphology—thus leading to a deeper understanding of the role of protein aggregation and disease progression.

## Materials and Methods

### Preparation of Aβ Solution

The recombinant protein was purchased from Abcam (ab120301 and ab120479) and prepared according to previous reports.^10^ In brief, a 10% ammonium hydroxide solution was used to dissolve 0.5 mg/mL of protein before being prepared as aliquots in protein low-bind Eppendorf tubes, lyophilized, and stored at −20 °C. For protein aggregation experiments, 50 μl of 60 μm NaOH was used to dissolve pellets, vortexed, and the concentration was determined using the NanoDrop ND 1000 spectrometer at 280 nm, the absorption coefficient of 1490 M^–1^ cm^–1^, and molecular weights for Aβ-40 of 4330 kDa and 4515 kDa for Aβ-42. Solutions were then diluted to a total 1 μM concentration in PBS or synthetic human CSF (ion concentrations of 150 mM Na, 3.0 mM K, 1.4 mM Ca, 0.8 mM Mg, 1.0 mM P, and 155 mM Cl) and allowed to aggregate at 37 °C for 48 h and constantly shaken at 400 rpm. For coaggregated samples, solutions were diluted to 500 nM per protein for a total of 1 μM concentration in the test sample. Synthetic CSF does not contain neurotransmitters. No additional proteins were used in the synthetic CSF. The pH of the as-received (10 mL) of synthetic CSF was measured to be 6.6 using a Mettler Toledo pH meter.

### AFM Measurements

AFM imaging was performed using the Dimension Bruker Icon 3 microscope operated in tapping mode. A volume of 5 μL of Aβ incubated protein solution or CSF samples was deposited on gold thin films (∼100 nm of Au (111) on mica substrate) and allowed to air-dry for 48 h. The gold substrates were purchased from Phasis, Inc., Switzerland. For proteins aggregated in CSF with an (IRHUCSFSYT25 ML, Innovative Research) and BioIVT patient CSF samples, high salt content resulted in the need for gently washing of the excess salts using ultrapure water. AFM topographical images were recorded, followed by 1× rinse with 5 μL molecular grade water, which was immediately removed before allowing the sample to dry before further imaging. Height measurements of sample structures were analyzed using Nanoscope software (Bruker) after first-order image flattening.

### Procedure for Antibody Staining

A volume of 50 μL of aggregated protein or CSF was incubated at RT for 30 min in poly-d-lysine-coated Ibidi μ-Slide 18 Well Glass Bottom plates to allow proteins to attach to the surface. Samples were fixed at room temperature for 20 min in 4% paraformaldehyde in PBS, washed 3× with PBS, and blocked in BlockAid (B10710, Thermo Fisher) for 1 h. Both primary and secondary antibodies were diluted in 1% BSA in PBS (37525, Thermo Fisher) at a dilution of 1:500. Aβ-40 protein was detected using mouse antihuman Aβ-40 IgG1 (AB20068, Abcam), Aβ-42 using rabbit antihuman Aβ-42 (AB180965, Abcam), Due to the potential for cross-reactivity of Aβ-42 primary antibody with Aβ-40 protein, anti-Aβ40 was incubated with the sample for 16 h (overnight) at room temperature to saturate epitopes. The antibody mixture was removed, followed by 1 h of incubation with anti-Aβ-42. Primary antibodies were washed off 3× with 1% BSA in PBS before incubation with secondary antibodies antirabbit Alexa Fluor@647 (CTK0101, Chromotek) and antimouse IgG1 Alexa Fluor@568 (CTK0103, Chromotek) for 1 h at RT in the dark. Samples were washed again 3× with 1% BSA in PBS, followed by a final rinse with molecular grade purity water before being prepared for super-resolution microscopy.

### Fluorescence Microscopy and Image Quantification Procedure

Samples were sealed in Everspark 1.0 buffer (Idylle) and imaged using a Nikon N-STORM microscope (Nikon UK Ltd.) with an SR Apochromat TIRF 100X 1.49 NA oil immersion objective lens. For super-resolution, Stochastic Optical Reconstruction Microscopy (STORM) imaging, in order to prevent axial drift during image acquisition, a built-in piezo-electric focus-lock system (perfect focus system) was used. The laser excitation at 647 nm had a peak density (at 100% power) of 1.2 kW/cm^2^ and at 561 nm of 0.55 kW/cm^2^. The emission was collected and passed through a QUAD filter set for TIRF applications (Nikon C–N STORM QUAD 405/488/561/647) comprising laser cleanup, dichroic and emission filters. Fluorescence was detected with an sCMOS Hamamatsu Orca Flash 4 v3, with an exposure time of 20 ms. Samples were first excited by the 647 nm laser (for Aβ-42), followed by the 561 nm laser (for Aβ-40). 10,000 frames were acquired per image, or a minimum of 4,000 if sample bleached before completion, which was more common for smaller aggregates. For patient CSF samples, single images were taken at 10–20% laser power. Images were processed on ImageJ using the Thunderstorm plugin as described below

### STORM Data Analysis

For STORM data the localizations were fitted with the integrated Gaussian point spread function in Thunderstorm,^63^ drift-corrected with cross correlation, and filtered with a cutoff in localization precision of 30 nm (for *x*–*y*) as well as a cut off in sigma (fwhm of the point spread function) of 200 nm. The reconstructed, super resolved pixel size was 8 nm. Area fraction of image was measured in ImageJ to quantify the amount of signal from Aβ-40 and Aβ-42 super-resolution images, respectively. This was used to indicate the aggregation and colocalization of proteins. First, grayscale images were adjusted to establish threshold of signal to measure using the same foreground channel settings for each area imaged, initially determined by the auto threshold feature, and adjusted if necessary. Area fraction was then measured to give a percentage of the image taken up by signal from each channel per image.

### Aggregation Kinetics Measurements Using Assays

ThT was diluted to 8uM in PBS or CSF. A volume of 200 μL was added to wells for triplicates of each sample and triplicate blanks for PBS and CSF containing ThT. Protein was prepared as described above and diluted to 500 nM (coaggregated samples) and 1 μM concentrations into wells after, and measurements were started immediately. An excitation wavelength of 440 ± 10 nm was used and emission was measured at 482 ± 20 nm and plates were not shaken with readings taken every 5 min for a minimum of 48 h at 37 °C.

### DHTM Measurements

Label-free holo-tomographic imaging was performed using a 3D Cell Explorer microscope (Nanolive SA, Switzerland). For each condition, 250 μL of 1 μM protein aggregates in solution was transferred to a 35 mm Ibidi uncoated μ-Dish (Ibidi GmbH, Germany) for imaging. Before each measurement, the Petri dish containing the protein aggregates in solution was placed in the microscope sample holder, and aggregates were allowed to sediment to the bottom of the Petri dish for 10 min before imaging. Each image acquired with the digital holo-tomographic microscope corresponds to a field of view of 90 μm × 90 μm × 30 μm. DHTM was operated under standard laboratory conditions. 3D RI stacks obtained by DHTM were exported as TIFF files and imported into the open-source software Tomviz for 3D RI visualization. The exported TIFF files were also imported into Imaris 9.8 (Bitplane AG, Switzerland) to achieve 3D surface segmentation. A surface was fitted with absolute intensity and automatic thresholding to achieve accurate signal segmentation and 3D rendering. For the structural analysis and quantification of the protein aggregates, 3D RI stacks obtained by DHTM were imported into the open-source software FIJI and a maximum intensity Z-projection was applied. The 2D images were exported as TIFF files and processed using a combination of Ilastik^51^ and Python-based analysis. First, the 2D images were loaded into Ilastik, an interactive machine-learning-based image segmentation tool. A pixel classification workflow was trained to differentiate between protein aggregates and backgrounds using manually labeled training data. Following segmentation, a probability map was generated and exported as an HDF5 file. Further downstream analysis was performed using Python in a Jupyter Notebook environment. The probability mask was thresholded to create a binary mask. Each object was labeled, and morphological properties were extracted using scikit-image’s regionprops function. The major axis length of each segmented object, corresponding to the longest dimension of the protein aggregate, was computed from the fitted ellipse. Additional filtering steps were applied to exclude artifacts based on object size and shape constraints.

### CSF Sample Preparation from AD Patients

Cerebrospinal fluid (CSF) samples were obtained from BioIVT, with 1 mL per patient ordered from their Alzheimer’s disease (AD) cohort. Samples were selected to ensure representation of both sexes and a range of ethnicities. Upon arrival, samples were aliquoted into 50 μL portions and stored at −80 °C for long-term storage. Patient samples were analyzed and characterized by BioIVT. Full details on the clinical evaluation of the patients (diagnosis, patient age, gender, ethnicity, and CSF analysis using biochemical assays) obtained from BioIVT are provided in Figure 5A. The AFM and fluorescence imaging on CSF samples from AD patients were conducted under identical protocols as employed for the characterization of protein aggregates in PBS solution and synthetic CSF.

All modeling system preparation steps, molecular dynamics simulation parameters, and detailed descriptions of the analyses conducted are provided in the Supporting Information section titled Molecular Modeling and Dynamics Simulations.

## Supplementary Material



## Data Availability

All MD data is deposited under Zenodo DOI: 10.5281/zenodo.16881329.

## Notes

AFM imaging in CSF and FTIR spectroscopy of Aβ-40 and Aβ-42 protein aggregates; details on computational model development and substrate-free simulations of coaggregated Aβ-40 and Aβ-42 in CSF (PDF)
