# Supplementary material for: Differentiating Alzheimer’s Aβ Isoforms Coaggregated in Cerebrospinal Fluid via Single-Particle Imaging
Source: ACS Chem Neurosci. 2026 Jan 14;17(3):550–64. doi: 10.1021/acschemneuro.5c00692 (PMC12879737; doi:10.1021/acschemneuro.5c00692)
Supplement: Supplementary file 1 [file cn5c00692_si_001.pdf]

Differentiating Alzheimer's Aβ isoforms co-aggregated in cerebrospinal fluid via single-particle imaging.

Lily Henry[1], Shayon Bhattacharya[2], Talia Bergaglio[1], Dorothea Pinotsi[3], and Peter Niraj Nirmalraj[1]

[1]Transport at Nanoscale Interfaces Laboratory, Swiss Federal Laboratories for Materials Science and Technology, Dübendorf, CH-8600, Switzerland. [2] Department of Biological Sciences, Bernal Institute, University of Limerick, Limerick V94T9PX, Ireland. [3] Scientific Centre for Optical and Electron Microscopy, ETH Zurich, Zürich, Switzerland.

Contents

Fig. S1: Schematic of indirect immunolabelling using nano secondary antibodies

Fig. S2: FTIR absorbance plots of Aβ-40 and Aβ-42 aggregated separately and co-aggregated in PBS and CSF

Fig. S3: AFM imaging of Aβ-40 aggregated independently in CSF

Fig. S4: Computational model development and simulation setup for Aβ-40 - Aβ-42 co-aggregates in PBS and CSF

Fig. S5: Substrate-free MD simulations of $Aβ_{40}$–$Aβ_{42}$ co-aggregation in CSF and comparison with substrate-supported systems

Fig. S6: AFM analysis of protofibril structure seen in patient 8822

Fig. S7: AFM and fluorescence analysis of additional Alzheimer's disease patient-derived CSF samples

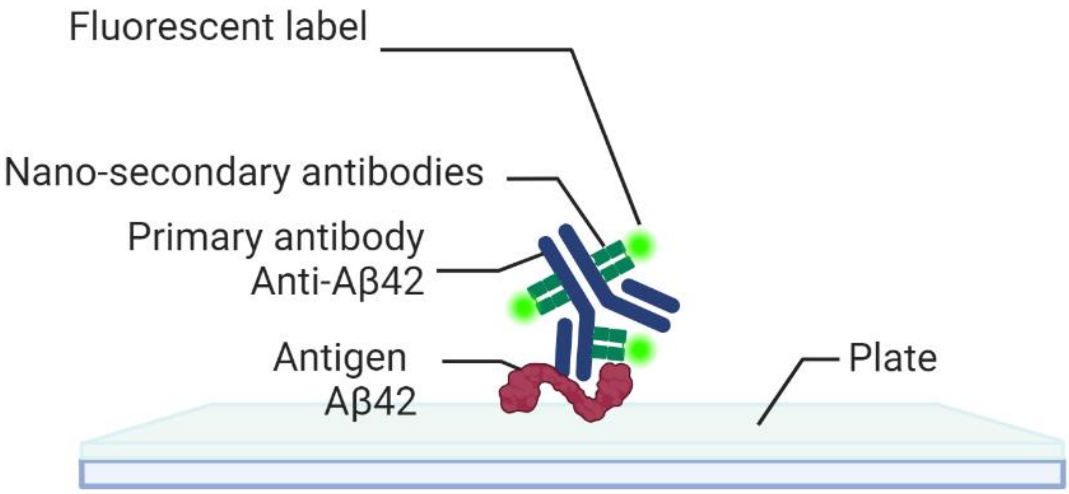

Fig. S1: Schematic of indirect immunolabelling using nano secondary antibodies. Highly specific primary antibodies bind to Aβ proteins, followed by subsequent binding of nano secondary antibodies. The use of nano secondary antibodies means closer proximity of the fluorophores to the proteins for increased resolution.

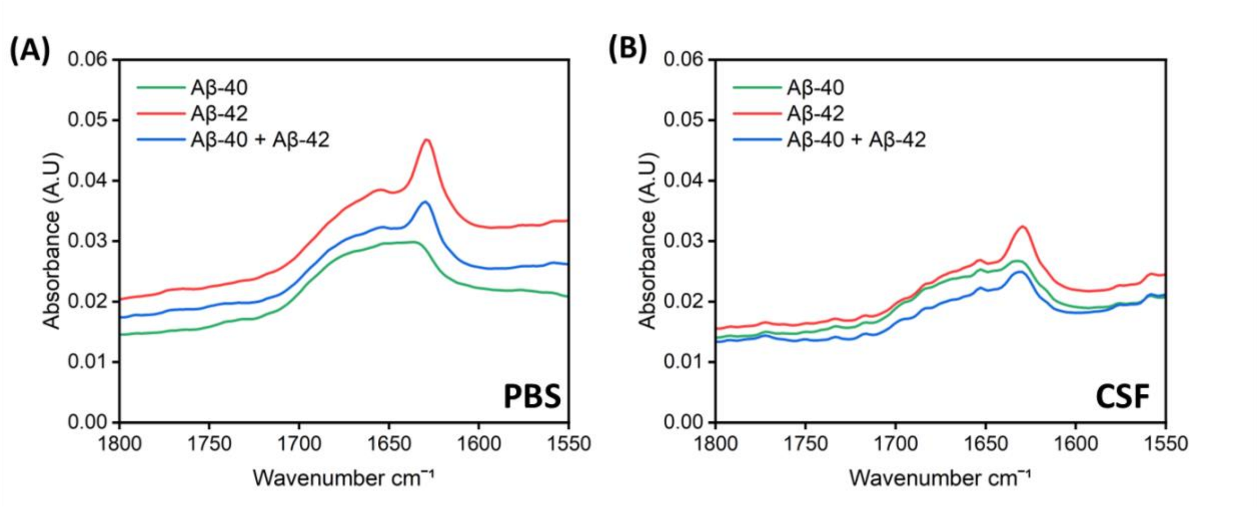

Fig. S2: FTIR absorbance plots of Aβ-40 and Aβ-42 aggregated separately and co-aggregated in PBS and CSF. (A) Comparison of absorption peaks for of Aβ-40 (green) and Aβ-42 (red) and co-aggregated Aβ-40 and Aβ-42 (blue) in PBS, showing characteristic beta-sheet absorption peak around 1630 cm^{-1} for Aβ-42 containing samples and absent in separately aggregated Aβ-40 sample. (B) Comparison of absorption peaks for of Aβ-40 (green) and Aβ-42 (red) and co aggregated Aβ-40 and Aβ-42 (blue) in CSF showing beta-sheet absorption peak around 1630 cm^{-1} for all samples, with a modest peak observed for Aβ-40.

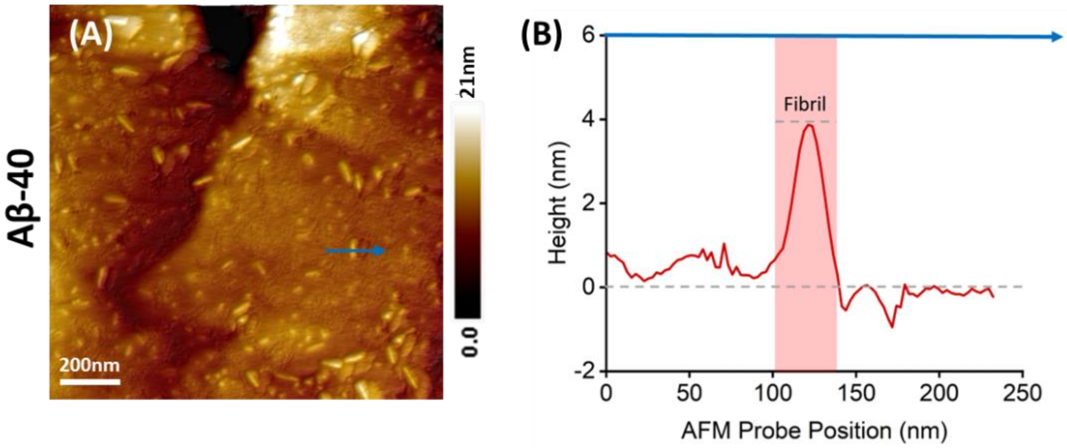

Fig. S3: AFM imaging of Aβ-40 aggregated individually in CSF. (A) AFM topographic image showing. (B) Cross-sectional height profile indicated by the blue arrow in panel A.

Molecular Modelling and Dynamics Simulations

Structure Preparation and Docking

To construct the co-aggregated Aβ complex, we used NMR-resolved and cryo-EM fibril structures of Aβ-40 and Aβ-42, respectively. A three-fold nonamer (trimer on each fold) of Aβ-40 (PDB ID: 2LMP[67]) was used to model the oligomeric component, while a two-fold 24-mer (AD-relevant dodecamer on each fold) of Aβ-42 (PDB ID: 2NAO[68]) was assembled as the fibrillar template. The Aβ-40 oligomer was docked onto the fibril using the ZDOCK[69] protocol, generating ten top-scoring poses ranked by energy. Among these, Model 2 was selected for molecular dynamics (MD) simulations as it best replicated the lateral surface deposition of oligomers observed in AFM and STORM experiments (Fig. S6A). In contrast, Model 1, which scored comparably in docking energy to Model 2, positioned the $A\beta_{40}$ oligomer along the fibril elongation axis — a configuration not supported by experimental AFM data and less consistent with known lateral association modes observed in physiological aggregation. Given their energetic similarity, Model 2 was selected as the more plausible starting structure based on structural compatibility with experimentally observed surface-bound co-aggregation patterns.

System Preparation

The selected Aβ-40/Aβ-42 co-aggregate was positioned atop an atomically flat gold substrate to mimic the experimental setup. The gold surface was modeled as a two-layer 15 × 15 nm^2 Au(111) slab with 0.3 nm thickness, held rigid throughout the simulations. The Aβ-42 fibril was oriented parallel to the surface, with its lateral axis initially placed ~6 Å above the gold plane. Simulations were conducted in both phosphate-buffered saline (PBS) and artificial cerebrospinal fluid (CSF), each comprising explicit solvent and ion environments matched to their respective experimental compositions . The CHARMM36m force field was used for proteins and gold, with water modeled using the CHARMM-modified TIP3P model. Ions were modelled using standard CHARMM parameters, and overall charge neutrality was maintained *via* background counterions.

MD Simulation Protocol

All simulations were performed using GROMACS 2018.4[70,71]. Systems were energy minimized using steepest descent, gradually heated to 298 K over 100 ps in the NVT ensemble, and equilibrated for 1 ns in

NVT and an additional 1 ns in NPT ensemble using the Berendsen[72] barostat (1 bar, time constant 4 ps). Temperature coupling was applied separately to protein and non-protein components using the velocity rescaling thermostat[73]. Bond constraints on proteins were applied using LINCS[74]; water hydrogens were constrained using SETTLE[75]. Long-range electrostatics were treated using the Particle Mesh Ewald (PME)[76] method with a 1.2 nm cutoff for nonbonded interactions. Production MD runs were carried out for 300 ns for each system under constant pressure using Parrinello-Rahman barostat[77] and temperature, with snapshots saved every 10 ps. To assess whether the gold substrate influenced the observed co-aggregation mechanism, we performed an additional 300-ns control simulation of $A\beta_{40}$–$A\beta_{42}$ in CSF without the Au(111) surface. All analyses (RMSD, hydrogen bonding, oligomer–fibril interaction energy, and ion–protein interaction profiles; Fig. S5C–G) confirm that Ca^{2+}- and Mg^{2+}-mediated contacts occur independently of the surface.

Trajectory Analysis

Trajectory analyses were conducted using GROMACS tools and custom Tcl/VMD scripts. Structural stability was evaluated by calculating the fraction of native contacts[78] (Q(X) values) relative to the initial docked conformation:

$$Q(X) \;=\; (1/N)\sum(i<j)\;\delta(r_{\mathrm{ij}}(X), r_{\mathrm{ij}}{}^{0}) \qquad \text{(Eq S1)}$$

where, $Q(X)$ is the fraction of native contacts at conformation X, N is the total number of native contacts in the reference structure, $r_{\mathrm{ij}}(X)$ is the distance between atoms i and j in conformation X, $r_{\mathrm{ij}}{}^{0}$ is the corresponding distance in the reference structure, and $\delta(r_{\mathrm{ij}}(X), r_{\mathrm{ij}}{}^{0} = 1$ if $\left|\delta(r_{\mathrm{ij}}(X) - r_{\mathrm{ij}}{}^{0}\right| < \varepsilon$, else 0.

Maximum height profiles of the co-aggregates relative to the gold surface were computed from trajectory frames using VMD scripting. Domain-resolved interaction energies between N-terminal, central hydrophobic core (CHC), and C-terminal regions of Aβ-40 and Aβ-42 were decomposed using energy group analysis over the final 20 ns of each trajectory. All energy values reported represent time-averaged values over the final 20 ns of simulation. The radius of gyration (Rg) of the Aβ-40 oligomer was extracted to assess compactness across solvent conditions.

To further quantify structural compaction and shape symmetry, the asphericity (Δ) of the Aβ-40 oligomer was calculated from the eigenvalues of the gyration tensor $\lambda 1, \lambda 2, \lambda 3$ as:

$$\Delta = \frac{1}{2} \times \frac{(\lambda 1-\lambda 2)^2+(\lambda 2-\lambda 3)^2+(\lambda 3-\lambda 1)^2}{(\lambda 1+\lambda 2+\lambda 3)^2} \qquad \text{(Eq S2)}$$

Here, Δ approaches zero for spherical shapes and increases with anisotropy. These calculations were performed over all heavy atoms of the Aβ-40 oligomer and averaged across the final 100 ns of the trajectory.

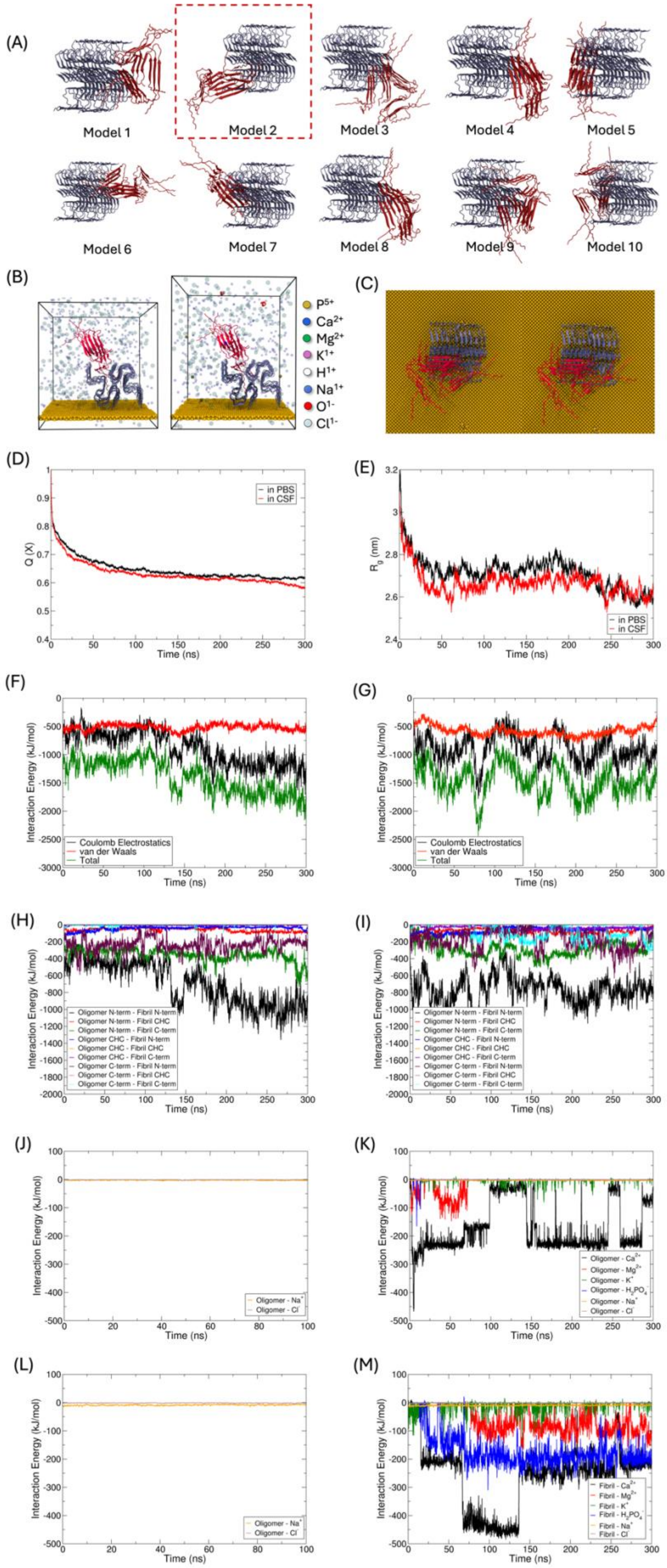

(A)
Model 1
Model 2
Model 3
Model 4
Model 5
Model 6
Model 7
Model 8
Model 9
Model 10
(B)
P5+
Ca2+
Mg2+
K1+
H1+
Na1+
O1-
Cl1-
(C)
(D)
Q (X)
Time (ns)
in PBS
in CSF
(E)
Rg (nm)
Time (ns)
in PBS
in CSF
(F)
Interaction Energy (kJ/mol)
Time (ns)
Coulomb Electrostatics
van der Waals
Total
(G)
Interaction Energy (kJ/mol)
Time (ns)
Coulomb Electrostatics
van der Waals
Total
(H)
Interaction Energy (kJ/mol)
Time (ns)
Oligomer N-term - Fibril N-term
Oligomer N-term - Fibril CHC
Oligomer N-term - Fibril C-term
Oligomer CHC - Fibril N-term
Oligomer CHC - Fibril CHC
Oligomer CHC - Fibril C-term
Oligomer C-term - Fibril N-term
Oligomer C-term - Fibril CHC
Oligomer C-term - Fibril C-term
(I)
Interaction Energy (kJ/mol)
Time (ns)
Oligomer N-term - Fibril N-term
Oligomer N-term - Fibril CHC
Oligomer N-term - Fibril C-term
Oligomer CHC - Fibril N-term
Oligomer CHC - Fibril CHC
Oligomer CHC - Fibril C-term
Oligomer C-term - Fibril N-term
Oligomer C-term - Fibril CHC
Oligomer C-term - Fibril C-term
(J)
Interaction Energy (kJ/mol)
Time (ns)
Oligomer - Na+
Oligomer - Cl-
(K)
Interaction Energy (kJ/mol)
Time (ns)
Oligomer - Ca2+
Oligomer - Mg2+
Oligomer - K+
Oligomer - H2PO4-
Oligomer - Na+
Oligomer - Cl-
(L)
Interaction Energy (kJ/mol)
Time (ns)
Oligomer - Na+
Oligomer - Cl-
(M)
Interaction Energy (kJ/mol)
Time (ns)
Fibril - Ca2+
Fibril - Mg2+
Fibril - K+
Fibril - H2PO4-
Fibril - Na+
Fibril - Cl-

Figure S4. Simulation setup, model selection, convergence analysis, and time-resolved energetics of Aβ$_{40}$–Aβ$_{42}$ co-aggregates. (A) Ten docked configurations of Aβ-40 nonamer on Aβ-42 fibril from ZDOCK screening. Model 2 (highlighted) was selected for simulations based on lateral binding consistent with experimental AFM images. (B) Simulation boxes for PBS and CSF systems showing explicit solvation, ion distribution, and complex placement on Au(111). (C) Top-view illustrations of complex placement on the gold substrate. (D) Fraction of native contacts Q(X) of the full complex (oligomer + fibril) as a function of time, showing convergence for both PBS and CSF trajectories over 300 ns. (E) Time evolution of radius of gyration (Rg) of the Aβ-40 oligomer. (F–G) Time-resolved oligomer–fibril interaction energies in PBS (F) and CSF (G), decomposed into Coulomb electrostatics, van der Waals, and total contributions. (H–I) Domain-specific oligomer–fibril interaction energy traces in PBS (H) and CSF (I), showing distinct temporal binding dynamics across N-term, CHC, and C-term. (J–K) Oligomer–ion interaction energies (normalized per ion) for all ionic species in PBS (J) and CSF (K), highlighting selective Ca^{2+} binding in CSF. (L–M) Fibril–ion interaction energies in PBS (L) and CSF (M), supporting the role of Ca^{2+} ions in stabilising the co-aggregate interface under CSF conditions.

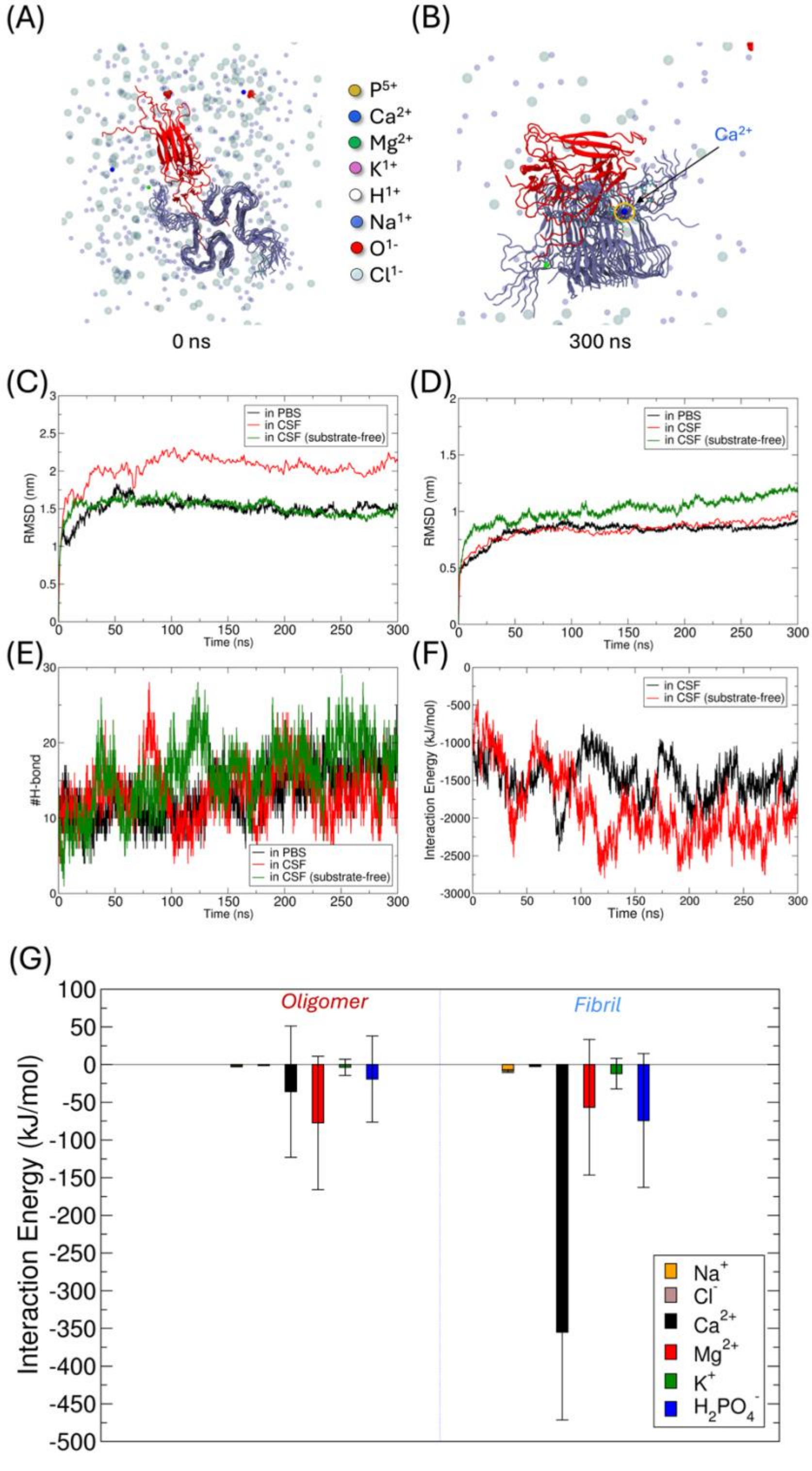

Figure S5. Substrate-free MD simulations of Aβ₄₀–Aβ₄₂ co-aggregation in CSF and comparison with substrate-supported systems. (A) Initial conformation (0 ns) of the $A\beta_{40}$ oligomer (blue) and $A\beta_{42}$ fibril fragment (red) in the CSF-like ionic environment without the Au(111) surface. All CSF ions are shown as spheres (legend inset). (B) Final conformation at 300 ns illustrating transient Ca^{2+}-mediated contacts between acidic residues on $A\beta_{40}$ and $A\beta_{42}$ (example Ca^{2+} bridge highlighted). (C–D) Backbone RMSD of the $A\beta_{40}$ oligomer (C) and $A\beta_{42}$ fibril fragment (D) over 300 ns for three systems: PBS (black), CSF on Au(111) (red), and CSF without substrate (green). (E) Total number of inter-protein hydrogen bonds between oligomer and

fibril across all three systems. (F) Time evolution of non-bonded interaction energy between oligomer and fibril in CSF with (red) and without (black) the Au(111) surface. (G) Average interaction energies between individual CSF ions and the Aβ_{40} oligomer (left) or Aβ_{42} fibril (right) in the substrate-free CSF simulation, showing predominant contributions from Ca^{2+} and Mg^{2+}, with Ca^{2+} exhibiting the strongest ion-mediated association consistent with transient Glu/Asp–Ca^{2+}–Glu/Asp bridging.

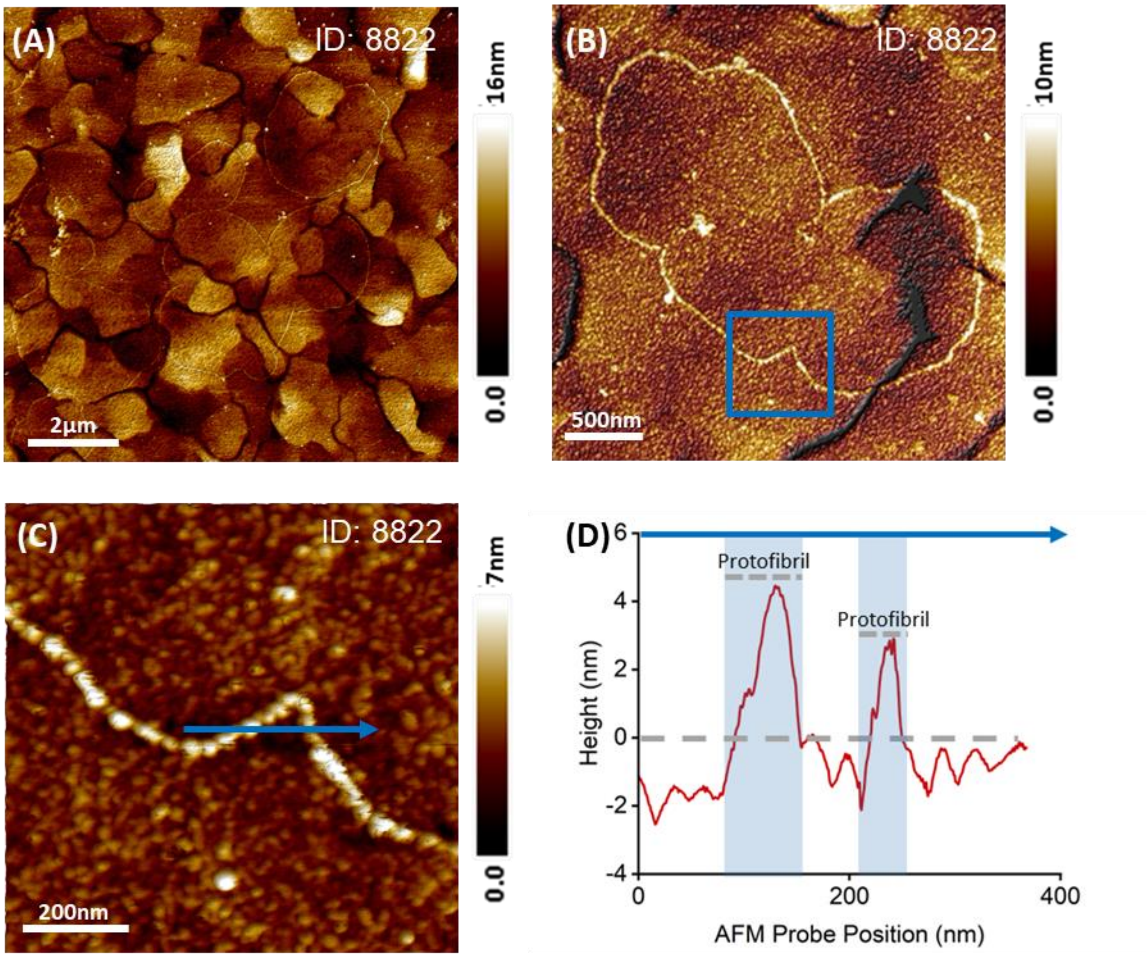

Figure S6: AFM analysis of ultralong protofibrils from patient 8822. (A) Large area AFM topographic image of ultralong protofibrils adsorbed on a gold substrate. (B) AFM topographical image of a single protofibril surrounded by spherical particles on a gold substrate. (C) High-resolution AFM topographical image within the region indicated by the blue box in (B). (D) The cross-sectional height profile is indicated by the blue arrow in (C).

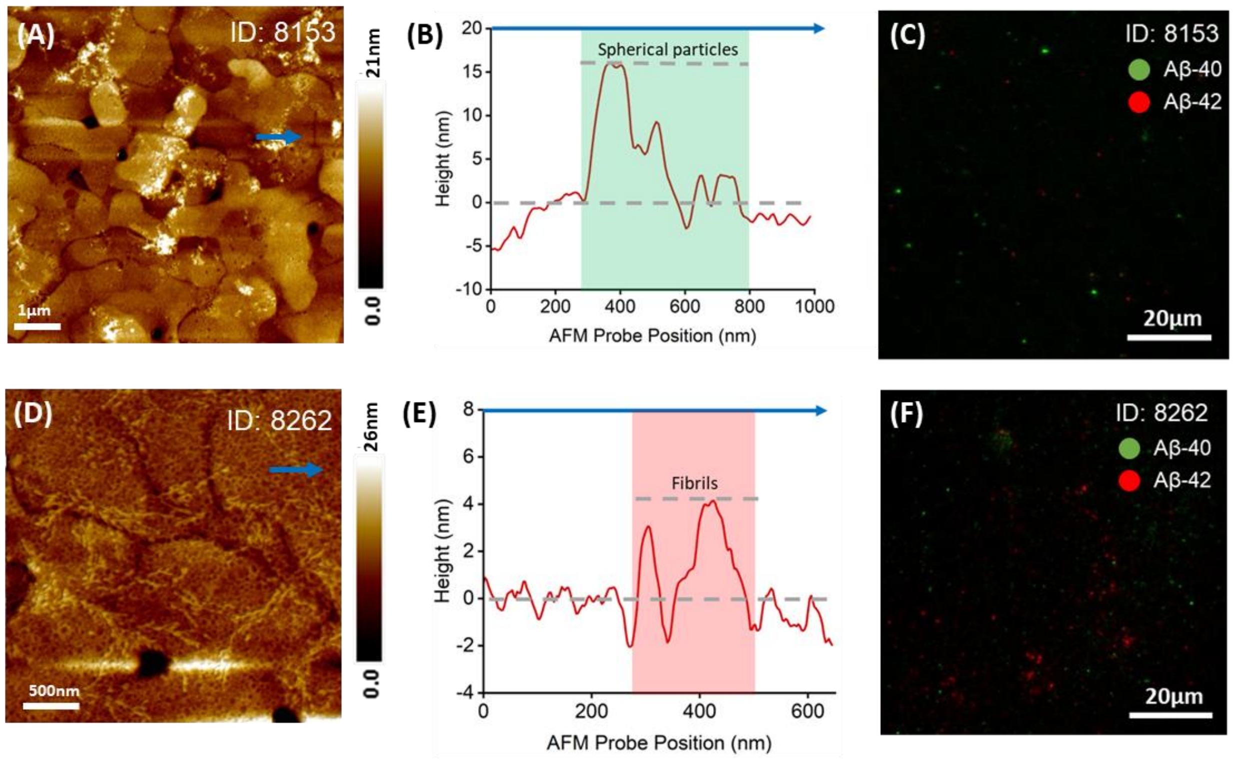

(G)

Patient ID: 8153		Patient ID: 8262	
Spherical particle clusters	12.5 ± 5.0 nm	Spherical particles	4.8 ± 2.1 nm
		Fibrils	3.6 ± 0.9 nm

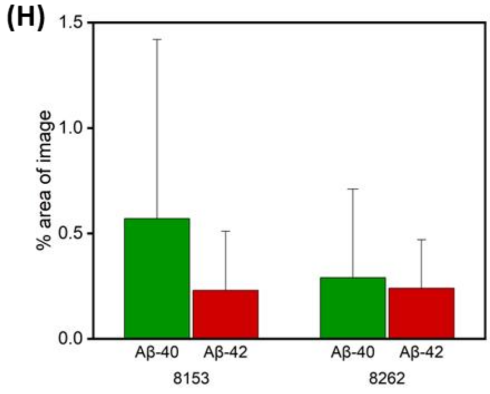

Figure S7: AFM and fluorescence analysis of additional Alzheimer's disease patient-derived CSF samples. (A) AFM topographic image of spherical particle clusters adsorbed on a gold substrate for patient 8153. (B) The cross-sectional height profile is indicated by the blue arrow in (A). (C) Widefield fluorescence image of Aβ-40 labelled with Alexa Fluor 561 in green and Aβ-42 labelled with Alexa Fluor 647 in red for patient 8153. (D) AFM topographical image of fibrils adsorbed on gold substrate for patient 8262. (E) Cross-sectional height profile is indicated by blue arrow in (D). (F) Widefield fluorescence image of Aβ-40 labelled with Alexa Fluor 561 in green and Aβ-42 labelled with Alexa Fluor 647 in red for patient 8262. (G) Quantification of aggregates identified in AFM topographical images: Patient ID: 8153 spherical particle clusters (n = 100, mean = 12.5 ± 5.0 nm), Patient ID: 8262 spherical particles (n = 106, mean = 4.8 ± 2.1 nm) and fibrils (n = 103, mean = 3.6 ± 0.9 nm). (H) Percentage area and standard deviation of image taken up from signal in each channel in 3 images of each patient sample: Patient ID: 8153 Aβ-40 (mean = 0.57 ± 0.85 %) and Aβ-42 (mean = 0.23 ± 0.28 %), Patient ID: 8262 Aβ-40 (mean = 0.29 ± 0.42 %) and Aβ-42 (mean = 0.24 ± 0.23 %).
